# BRCA1 and CtIP promote alternative non-homologous end-joining at uncapped telomeres

**DOI:** 10.15252/embj.201488947

**Published:** 2015-01-12

**Authors:** Sophie Badie, Ana Rita Carlos, Cecilia Folio, Keiji Okamoto, Peter Bouwman, Jos Jonkers, Madalena Tarsounas

**Affiliations:** 1 Telomere and Genome Stability Group, The CR-UK/MRC Gray Institute for Radiation Oncology and BiologyOxford, UK; 2 Department of Molecular and Experimental Medicine, The Scripps Research InstituteLa Jolla, CA, USA; 3 Division of Molecular Pathology and Cancer Systems Biology Centre, The Netherlands Cancer InstituteAmsterdam, The Netherlands

**Keywords:** alternative non-homologous end-joining, BRCA1/CtIP, telomere, TRF2

## Abstract

Loss of telomere protection occurs during physiological cell senescence and ageing, due to attrition of telomeric repeats and insufficient retention of the telomere-binding factor TRF2. Subsequently formed telomere fusions trigger rampant genomic instability leading to cell death or tumorigenesis. Mechanistically, telomere fusions require either the classical non-homologous end-joining (C-NHEJ) pathway dependent on Ku70/80 and LIG4, or the alternative non-homologous end-joining (A-NHEJ), which relies on PARP1 and LIG3. Here, we show that the tumour suppressor BRCA1, together with its interacting partner CtIP, both acting in end resection, also promotes end-joining of uncapped telomeres. BRCA1 and CtIP do not function in the ATM-dependent telomere damage signalling, nor in telomere overhang removal, which are critical for telomere fusions by C-NHEJ. Instead, BRCA1 and CtIP act in the same pathway as LIG3 to promote joining of de-protected telomeres by A-NHEJ. Our work therefore ascribes novel roles for BRCA1 and CtIP in end-processing and fusion reactions at uncapped telomeres, underlining the complexity of DNA repair pathways that act at chromosome ends lacking protective structures. Moreover, A-NHEJ provides a mechanism of previously unanticipated significance in telomere dysfunction-induced genome instability.

## Introduction

Telomeres are structures at the ends of linear chromosomes, which consist of long stretches of repetitive double-stranded DNA ending in a 3′ single-stranded overhang. Shelterin, a specialized protein complex which binds telomeric DNA with sequence specificity, is critical for telomere homeostasis and their distinction from unrepaired double-strand breaks (DSBs). In addition, telomeres are bound by factors involved in chromatin remodelling and DNA damage repair. Interestingly, several DNA damage response (DDR) factors are required for proper telomere maintenance, drawing a thin line between telomere protection and their recognition as broken DNA ends.

The shelterin component TRF2 plays a key role in telomere protection and prevents ATM-dependent DNA damage signalling at telomeres. Loss of TRF2 elicits telomere uncapping which in turn activates checkpoint responses visualized through accumulation of DDR factors [e.g. phosphorylated histone H2AX (γH2AX), MRE11, phosphorylated ATM, 53BP1] at telomeres, into microscopically defined telomere dysfunction-induced foci (TIFs; di Fagagna *et al*, 2003; Takai *et al*, 2003). In normal cells, the DDR emanating from uncapped telomeres leads to irreversible cell cycle arrest or cell death, thus limiting the proliferative potential of cells harbouring potentially deleterious mutations. This phenotype is similar to the state arising during physiological ageing and senescence due to erosion of telomeric repeats. Alternatively, telomeric DDR activates downstream cell cycle effectors, which engage the DNA repair machinery to join unprotected telomeres into aberrant chromosomal end-to-end fusions that drive genomic instability and oncogenic transformation (Maser & DePinho, 2004).

Fusions of telomeres uncapped through TRF2 deletion require primarily classical NHEJ (C-NHEJ) reactions, dependent on Ku70/80 and the DNA ligase 4 (LIG4: Celli & de Lange, 2005; Celli *et al*, 2006). In addition, telomeric joining can be mediated by A-NHEJ (Rai *et al*, 2010; Sfeir & de Lange, 2012), an end-joining pathway related to C-NHEJ, but known to be Ku and LIG4 independent (Feldmann *et al*, 2000; Guirouilh-Barbat *et al*, 2004). A-NHEJ reactions have been implicated in DSB repair and V(D)J recombination (Nussenzweig & Nussenzweig, 2007; McVey & Lee, 2008; Iliakis, 2009) and are mediated by DNA ligase 3 (LIG3) and poly (adenosine diphosphate ribose) polymerase 1 (PARP1: Audebert *et al*, 2004; Wang *et al*, 2006; Mansour *et al*, 2010). Mechanistically, A-NHEJ repair requires limited resection of DNA ends (4–6 nucleotides) to generate short complementary sequences that stabilize end ligation. At intrachromosomal DSBs, this process involves the MRE11-RAD50-NBS1 (MRN) complex and CtIP (Bennardo *et al*, 2008; Rass *et al*, 2009; Xie *et al*, 2009). Moreover, the end resection activity of CtIP promotes A-NHEJ reactions leading to chromosomal translocations (Zhang & Jasin, 2011).

The tumour suppressor BRCA1 functions in homologous recombination repair to activate CtIP through ubiquitylation (Yu *et al*, 2006), which mediates its recruitment to sites of damage in cell cycle-regulated manner (Yun & Hiom, 2009). CDK-dependent phosphorylation of CtIP during S/G2 promotes its interaction with the C-terminal BRCT domain of BRCA1 (Yu *et al*, 1998; Chen *et al*, 2008). A complex formed by BRCA1-MRN-CtIP has been reported to play a critical role in DSB resection required for recombinational repair of IR-induced DSB (Buis *et al*, 2008; Chen *et al*, 2008). Whilst the roles of MRN and CtIP in A-NHEJ repair are well established (You & Bailis, 2010), a similar function for BRCA1 has not yet been defined in spite of clear functional interactions between these factors in end resection.

Here, we employ telomeres artificially uncapped through TRF2 shRNA-mediated depletion in mouse embryonic fibroblasts (MEFs) to study the role of A-NHEJ in telomere end-joining reactions. Our system differs significantly from the complete removal of shelterin from chromosome ends triggered by *Trf1/2* gene deletions used in previous studies (Sfeir & de Lange, 2012). In TRF2-depleted cells, telomere architecture is only partially compromised, as shelterin components other than TRF2 and RAP1 remain associated with the telomeres. This results in DDR attenuation and limited end-processing reactions (e.g. resection), compared to shelterin-free ends. Using this system, we demonstrate that BRCA1 acts in the same pathway as CtIP, LIG3 and PARP1 to promote A-NHEJ at dysfunctional telomeres. Although the DNA damage signal emanating from uncapped telomeres is not affected by conditional *Brca1* deletion in MEFs, the frequency of end-to-end fusions is significantly reduced to levels similar to CtIP-, LIG3- or PARP1-depleted cells. This is likely due to inhibition of end-processing reactions required for A-NHEJ of uncapped telomeres, which also involves EXO1. Our study therefore assigns a key function to BRCA1 in A-NHEJ and defines novel roles for the end resection factors (BRCA1, CtIP and EXO1) in processing dysfunctional telomeres.

## Results

### BRCA1 and CtIP are not required for the DDR emanating from uncapped telomeres

TRF2 removal from the telomeres has two important consequences: activation of an ATM-dependent DDR and joining of uncapped telomeres with formation of end-to-end fusions (Palm & de Lange, 2008), both orchestrated by the MRN complex. Consistent with this, MRN inactivation under telomere dysfunction conditions (e.g. TRF2 depletion) abolishes telomeric accumulation of DDR factors and telomeric fusions (Attwooll *et al*, 2009; Deng *et al*, 2009; Dimitrova & de Lange, 2009). MRN interacts with CtIP and BRCA1 in cell cycle-regulated manner during the normal cellular response to DNA damage (Chen *et al*, 2008). To address whether CtIP and BRCA1 play similar roles to MRN at uncapped telomeres, we examined both the integrity of the DNA damage signalling pathways and the frequency of telomeric fusions in cells lacking CtIP or BRCA1.

**Figure 1:**
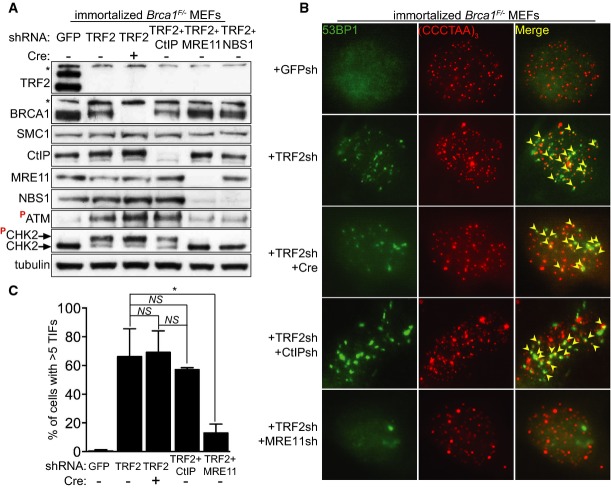
The DNA damage response at telomeres uncapped through TRF2 depletion does not require BRCA1 or CtIP
Immortalized *Brca1*^*F/−*^ MEFs were infected with retroviruses expressing the indicated shRNAs and/or Cre recombinase, followed by selection with puromycin for 72 h. Cell extracts were prepared 48 h later and analysed by Western blotting as indicated. SMC1 and tubulin were used as loading controls. *non-specific band.Cells treated as in (A) were fixed 48 h after selection and stained with an anti-53BP1 antibody (green). Telomeres were visualized with a Cy3-conjugated (CCCTAA)_3_-PNA probe (red). Yellow arrowheads point to 53BP1 foci that co-localize with telomeres.Quantification of TIFs in cells treated as in (B). A minimum of 200 nuclei were scored for each sample. Error bars represent SD of two independent experiments. *P*-values were calculated using an unpaired two-tailed *t-*test. *P ≤ 0.05; NS, *P *> 0.05.

To artificially induce loss of telomere protection, we inhibited expression of the shelterin component TRF2 using retrovirus-delivered short hairpin RNA (shRNA; Fig1A). As previously reported (Rai *et al*, 2010), this triggered a robust phosphorylation of the DNA damage checkpoint kinase CHK2 monitored by Western blotting, indicative of an ATM-dependent DNA damage response (Fig1A). To address the impact of BRCA1 on the DNA damage signal emanating from uncapped telomeres, we conditionally deleted *Brca1* using Cre and concomitantly depleted TRF2 in *Brca1*^*F/−*^ MEFs (Bouwman *et al*, 2010). In these cells, exons 5 and 6 of *Brca1* are flanked by loxP sites and cleavable upon treatment with self-inactivating (‘Hit&Run’) Cre recombinase (Silver & Livingston, 2001). BRCA1 expression was effectively abrogated, as shown in Western blot analyses (Fig1A). As *Brca1* is essential for cell survival, we immortalized the *Brca1*^*F/−*^ MEFs by stable expression of the p53 shRNA or SV40 large T antigen (LT), a suppressor of both pRb- and p53-dependent senescence pathways. The same MEFs were separately transduced with CtIP, MRE11 and NBS1 shRNAs, each in combination with TRF2 shRNA, and the reduction in protein levels was monitored by Western blotting. The robust CHK2 phosphorylation triggered by TRF2 inhibition was only suppressed by MRE11 or NBS1 depletion, but not by BRCA1 or CtIP abrogation (Fig1A). This suggested that BRCA1 or CtIP is not required for ATM-dependent signalling at uncapped telomeres.

Recruitment of DDR factors to telomeres uncapped through TRF2 depletion leads to TIF formation. 53BP1 is a DDR component known to associate with uncapped telomeres where it alters chromatin behaviour and promotes telomere fusions (Dimitrova *et al*, 2008). In cells treated with the same shRNAs as above, we quantified TIF formation by monitoring the co-localization of 53BP1 with the telomeric FISH signal (Fig1B). Consistent with previous studies (Attwooll *et al*, 2009; Deng *et al*, 2009), TIFs induced by TRF2 depletion in interphase cells decreased significantly (from approximately 66 to 13%) upon concomitant inhibition of MRE11 (Fig1C). In contrast, a similar analysis of BRCA1- or CtIP-deficient cells revealed no significant effect on TIF formation. These results suggest that, in spite of their physical interaction with the MRN complex, CtIP and BRCA1 are not required for the DNA damage signalling triggered by TRF2 abrogation.

### BRCA1 and CtIP are required for telomeric fusions in TRF2-depleted cells

In addition to ATM-dependent checkpoint signalling, end-to-end fusions are a hallmark of TRF2 inactivation (Palm & de Lange, 2008). The MRN complex mediates this process both through ATM activation and through the nucleolytic removal of the 3′ telomeric overhang (Deng *et al*, 2009). MRN-dependent nuclease activity is stimulated by CtIP (Sartori *et al*, 2007), which in turn requires BRCA1 for its activation. We therefore set to investigate whether CtIP and/or BRCA1 can facilitate fusion of TRF2-depleted telomeres.

**Figure 2:**
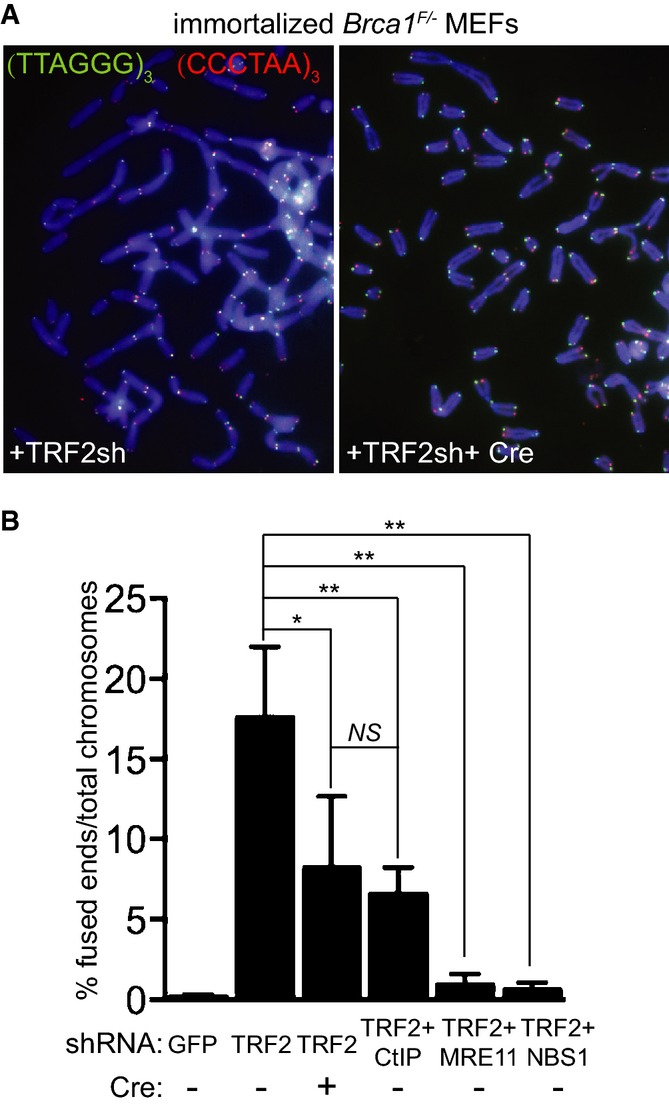
BRCA1 and CtIP are required for telomeric end-to-end fusions induced by TRF2 inactivation
Immortalized *Brca1*^*F/−*^ MEFs were infected with retroviruses expressing the indicated shRNAs and/or Cre recombinase, followed by selection with puromycin for 72 h. Cells were arrested in mitosis with colcemid and mitotic chromosomes were processed for CO-FISH analysis. Metaphase chromosome spreads were stained with Cy3-conjugated leading strand telomeric PNA probe (red) and FITC-conjugated lagging strand telomeric PNA probe (green). DNA was counter-stained with DAPI (blue).The frequency of chromosome-type telomeric fusions in cells treated as in (A) was quantified as a percentage of total number of chromosomes (illustrated in Supplementary Fig S2A). A minimum of 2,000 chromosomes were scored for each treatment. Error bars represent SD of at least two independent experiments. *P*-values were calculated using an unpaired two-tailed *t-*test. *P ≤ 0.05; **P ≤ 0.01; NS, *P *> 0.05.

To study the impact of BRCA1 or CtIP abrogation on telomeric fusions, we performed chromosome orientation fluorescence *in situ* hybridization (CO-FISH) on metaphase chromosomes isolated from *Brca1*^*F/−*^ MEFs treated with TRF2 shRNA, either alone or in combination with Cre recombinase, CtIP, MRE11 or NBS1 shRNAs (Fig2A). We observed a significant reduction in fusion number in cells treated with both Cre and TRF2 shRNA, relative to control cells treated with TRF2 shRNA alone. TRF2 depletion triggered fusion frequencies of approximately 17.6%, whilst concomitant *Brca1* deletion caused a reduction to 8.2% (Fig2B), in the same range as CtIP depletion (6.6%). Most likely, the residual fusions detected after TRF2/BRCA1 or TRF2/CtIP inhibition were mediated by C-NHEJ (see below). Statistical analyses showed that the decrease in fusion frequencies in TRF2-depleted cells also lacking BRCA1 or CtIP was significant when compared to cells lacking TRF2 alone (*P *≤ 0.05), indicating that these factors promote end-joining of uncapped telomeres. As controls, we measured fusion formation in TRF2-depleted MEFs treated with MRE11 or NBS1 shRNAs. The fusion frequencies obtained in this analysis (0.9 and 0.6%, respectively; Fig2B) were lower than those in CtIP-depleted cells. This reflects the multiple roles of MRN complex in the DNA damage response leading to telomeric fusions (Stracker & Petrini, 2011), including ATM recruitment and activation, as well as overhang cleavage to facilitate end-joining.

To verify that the observed reduction in fusion frequency was not a consequence of impaired cell proliferation or defects in cell cycle progression, we measured cell growth rates when TRF2 was inactivated alone or in combination with either BRCA1 or CtIP (Supplementary Fig S1A). We observed no significant effect on the proliferation of TRF2-depleted MEFs when BRCA1 or CtIP was additionally inactivated. Similarly, no significant differences in cell cycle progression were detected between these cell populations using FACS analyses (Supplementary Fig S1B), which indicated that the reduced fusion frequencies were not due to cell cycle arrest in a stage non-permissive for ligation to occur.

Upon inhibition of the MRN complex, formation of chromosome end-to-end fusions in TRF2-depleted MEFs was impaired. However, chromatid fusions involving the telomeric leading strand remained detectable using CO-FISH (Supplementary Fig S2A, B and D), consistent with the reported role for MRN in post-replicative processing of the telomeric leading strand (Attwooll *et al*, 2009; Deng *et al*, 2009; Dimitrova & de Lange, 2009). Interestingly, BRCA1 and CtIP abrogation also led to an increased frequency of chromatid fusions in TRF2-depleted cells (from 2.7 to 15.5% and 13.8%, respectively; Supplementary Fig S2C). CO-FISH staining revealed that chromatid fusions in BRCA1- and CtIP-deficient MEFs involved both the leading and the lagging strand of the telomeres, with a slight bias towards lagging–lagging fusions (Supplementary Fig S2D). This suggests a requirement for these factors in the post-replicative processing of both lagging and leading telomeric strands at uncapped telomeres.

### BRCA1 and CtIP act in a Ku80/LIG4-independent and PARP1/LIG3-dependent pathway for telomere fusion

**Figure 3:**
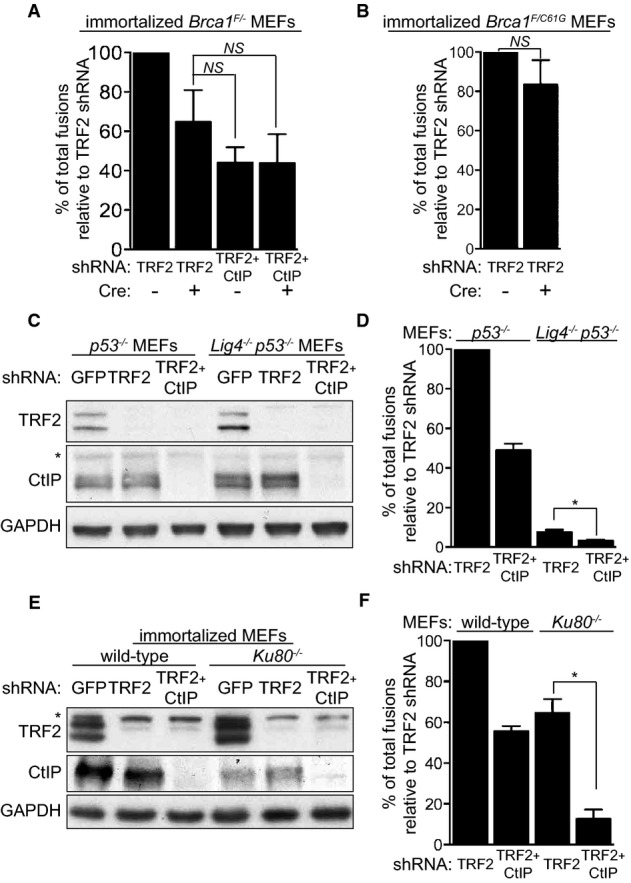
BRCA1 and CtIP mediate Ku- and LIG4-independent telomere fusions
Immortalized *Brca1*^*F/−*^ MEFs were infected with retroviruses expressing the indicated shRNAs and/or Cre recombinase, followed by selection with puromycin for 72 h. Mitotic chromosomes isolated 48 h later were fixed and stained with a Cy3-conjugated (CCCTAA)_3_-PNA probe. The frequency of end-to-end chromosome-type fusions is represented as a percentage of fusions observed after TRF2 depletion. A minimum of 2,000 chromosomes were scored for each sample. Error bars represent SD of three independent experiments. *P*-values were calculated using an unpaired two-tailed *t-*test. NS, *P *> 0.05.Immortalized *Brca1*^*F/C61G*^ MEFs were infected with retroviruses expressing the indicated shRNAs and/or Cre recombinase, followed by selection with puromycin for 72 h. The frequency of end-to-end chromosome-type fusions was analysed as in (A).MEFs of the indicated genotypes were infected with retroviruses expressing TRF2 and/or CtIP shRNAs, followed by selection with puromycin for 72 h. Cell extracts were prepared 48 h later and analysed by Western blotting as indicated. GAPDH was used as a loading control. *non-specific band. Cells treated as in (C) and (E) were arrested in mitosis with colcemid, and mitotic chromosomes isolated 48 h later were fixed and stained with a Cy3-conjugated (CCCTAA)_3_-PNA probe (D, F). The frequency of end-to-end chromosome-type fusions is represented as a percentage of fusions observed after TRF2 depletion. Error bars represent SD of two independent experiments. *P*-values were calculated using an unpaired two-tailed *t-*test. *P ≤ 0.05.

We observed that BRCA1 and CtIP deficiencies lower the number of fusions in TRF2-depleted cells to similar levels (Fig2B), thus raising the possibility that they act in the same pathway of uncapped telomere processing and re-joining. To address this possibility, we inactivated BRCA1 and CtIP concomitantly in TRF2-depleted cells. The reduction in telomeric fusions observed in the double BRCA1/CtIP-defective cells was comparable to cells in which BRCA1 or CtIP was separately inactivated (Fig3A), suggesting that they are epistatic to each other with respect to uncapped telomere processing. Importantly, the cancer-predisposing C61G mutation in BRCA1, which abrogates its E3 ubiquitin ligase activity, and disrupts the BRCA1 RING domain and its interaction with BARD1 (Drost *et al*, 2011), did not significantly alter the frequency of fusions induced by TRF2 depletion. This indicates that BRCA1 E3 ligase activity is dispensable for its function at dysfunctional telomeres.

C-NHEJ together with A-NHEJ mediates joining of uncapped telomeres in mammalian cells (Sfeir & de Lange, 2012). Consistent with LIG4-dependent canonical NHEJ being the primary mechanism for uncapped telomere ligation, we found that fusions elicited by TRF2 removal in *Lig4*^*−/−*^ MEFs (Fig3C) were lowered to approximately 7.6% compared to LIG4-proficient cells (Fig3D). Importantly, when CtIP was depleted together with TRF2 in *Lig4*^*−/−*^ MEFs, the fusion frequency was further reduced to approximately 3.5%, suggesting that CtIP acts in a LIG4-independent pathway of uncapped telomere fusion. An additive effect on fusion suppression was also observed when BRCA1 expression was inhibited using shRNAs in *Lig4*^*−/−*^ MEFs (Supplementary Fig S3), which indicates that BRCA1 also functions independently of LIG4 at uncapped telomeres. Although in *Ku80*^*−/−*^ MEFs the fusion frequency induced with TRF2 shRNA was reduced to approximately 64.7% relative to controls (Fig3E and F), a much higher value than in TRF2-depleted *Lig4*^*−/−*^ MEFs (7.6%), CtIP inhibition in these cells further decreased fusion rates to 12.7%. These differences in the capacity of *Ku80*^*−/−*^ and *Lig4*^*−/−*^ MEFs to re-join dysfunctional telomeres are most likely linked to their distinct functions in DSB repair at intra-chromosomal sites (see Discussion).

**Figure 4:**
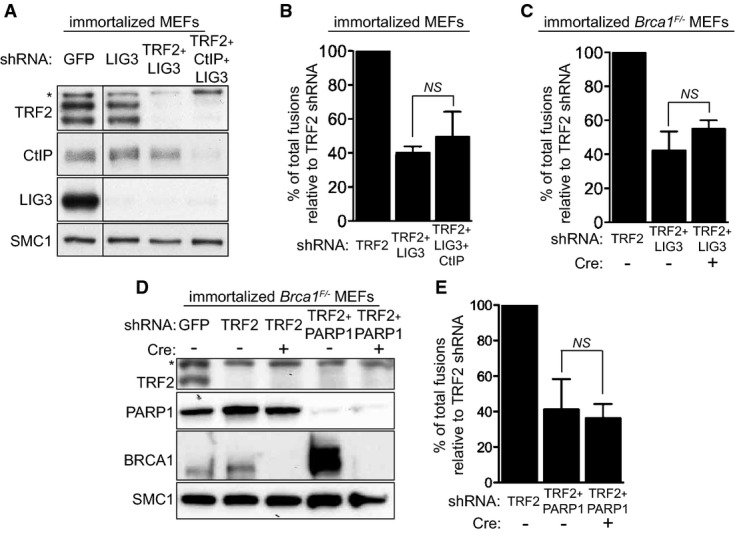
BRCA1 and CtIP promote LIG3- and PARP1-dependent end-joining of telomeres uncapped by TRF2 inactivation
Immortalized MEFs were infected with retroviruses expressing the indicated shRNAs, followed by selection with puromycin for 72 h. Cell extracts were prepared 48 h later and analysed by Western blotting as indicated. SMC1 was used as a loading control. *non-specific band.Quantification of the frequency of end-to-end chromosome-type fusions of cells treated as in (A) represented as a percentage of fusions observed after TRF2 depletion. A minimum of 2,000 chromosomes were scored for each sample. Error bars represent SD of three independent experiments. The *P*-value was calculated using an unpaired two-tailed *t-*test. NS, *P *> 0.05.Immortalized *Brca1*^*F/−*^ MEFs were infected with retroviruses expressing the indicated shRNAs and/or Cre recombinase. Mitotic chromosomes isolated 48 h later were fixed and stained with a Cy3-conjugated (CCCTAA)_3_-PNA probe. The frequency of end-to-end chromosome-type fusions is represented as a percentage of fusions observed after TRF2 depletion. A minimum of 2,000 chromosomes were scored for each sample. Error bars represent SD of three independent experiments. *P*-values were calculated using an unpaired two-tailed *t-*test. NS, *P *> 0.05.Immortalized *Brca1*^*F/−*^ MEFs were infected with retroviruses expressing TRF2 shRNAs and/or Cre recombinase, together with a lentivirus expressing PARP1 shRNA, followed by selection with puromycin for 72 h. Cell extracts were prepared 48 h later and analysed by Western blotting as indicated. SMC1 was used as a loading control. *non-specific band.Quantification of the frequency of end-to-end chromosome-type fusions of cells treated as in (D) represented as a percentage of fusions observed after TRF2 depletion. A minimum of 1,500 chromosomes were scored for each sample. Error bars represent SD of three independent experiments. The *P-*value was calculated using an unpaired two-tailed *t-*test. NS, *P *> 0.05.

A-NHEJ provides a mechanism for DSB repair that is Ku and LIG4 independent, relying instead on PARP1 and LIG3 for ligating DNA ends (reviewed in Nussenzweig & Nussenzweig, 2007; McVey & Lee, 2008; Iliakis, 2009). The fusion frequency in cells in which LIG3 was co-depleted together with TRF2 was reduced to approximately 40.1% of that of LIG3-proficient cells (Fig4A and B). This was surprising because only 7.6% of the fusions were anticipated to require A-NHEJ based on the effect of TRF2 depletion in *Lig4*^*−/−*^ MEFs (Fig3D). A possible explanation for this paradox is the complex interplay postulated between LIG3 and LIG4 in DNA repair (McVey & Lee, 2008), including the possibility that the two act together in the final ligation step of the reaction.

The role of CtIP in A-NHEJ at intra-chromosomal DSBs has received increasing experimental support in recent years (Bennardo *et al*, 2008; You & Bailis, 2010; Zhang & Jasin, 2011; Grabarz *et al*, 2013). To address whether CtIP acts in A-NHEJ at TRF2-depleted telomeres, we examined the impact of CtIP depletion on telomeric fusions in the context of LIG3 inactivation (Fig4B). Concomitant CtIP and LIG3 inhibition triggered a decrease in the number of fusions (to approximately 49.5%) comparable to that caused by LIG3 inactivation alone (approximately 40.1%). A similar result was obtained when *Brca1* was deleted using Cre treatment in cells lacking LIG3 expression (Fig4C). The reduction in telomeric fusions elicited by BRCA1 abrogation (approximately 55%) did not differ significantly from the effect of LIG3 inhibition alone (approximately 42.3%). Furthermore, we examined the effect of PARP1, a factor known to stimulate LIG3-dependent end-joining, on dysfunctional telomere ligation. Fusion frequencies were reduced to 41.6% in PARP1-depleted MEFs and to 36.3% when BRCA1 was additionally deleted (Fig4D and E), suggesting that PARP1 and BRCA1 are epistatic to each other with respect to telomere fusion formation. Taken together, these results demonstrate that BRCA1, CtIP, PARP1 and LIG3 act in the same pathway for re-joining dysfunctional telomeres.

### CtIP is not required for the removal of the 3′ overhang at uncapped telomeres

**Figure 5:**
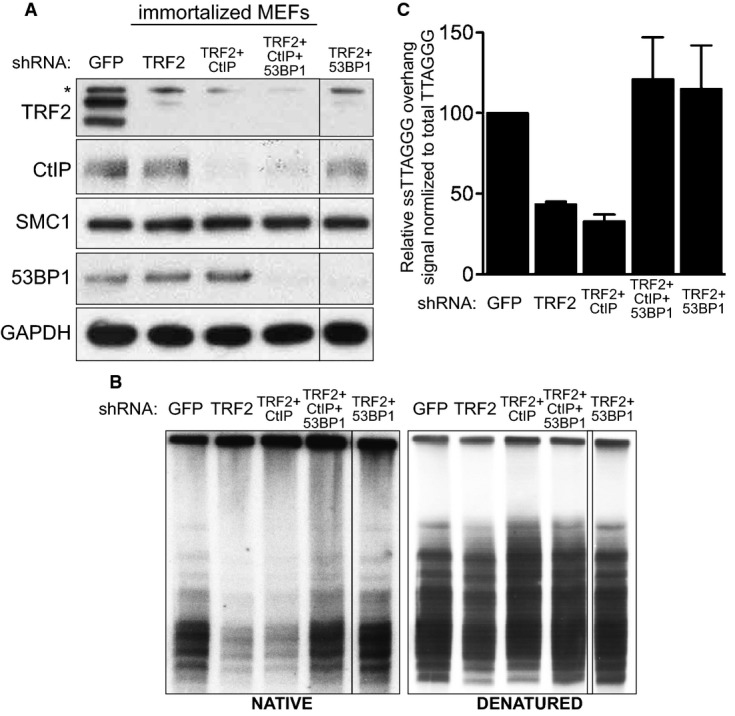
CtIP is not required for degradation of the single-stranded telomeric overhang generated by TRF2 inactivation
Immortalized MEFs were infected with retroviruses expressing the indicated shRNAs, followed by selection with puromycin for 72 h. Cell extracts were prepared 48 h later and analysed by Western blotting as indicated. SMC1 and GAPDH were used as loading controls. *non-specific band.MboI- and AluI-digested DNA from cells treated as in (A) was resolved by pulsed-field gel electrophoresis and probed with end-labelled (AACCCT)_4_ probe. Representative pulsed-field gel samples run under native and denatured conditions are shown.Quantification of the 3′ overhang in cells treated as in (B). For each sample, the ss/total DNA ratios were expressed relative to the GFP shRNA-treated control. Error bars represent SD of two independent experiments.

Telomeric fusions in cells lacking TRF2 require MRN-dependent nucleolytic removal of the 3′ overhang (Deng *et al*, 2009; Dimitrova & de Lange, 2009). Consistent with this, pulsed-field gel electrophoresis of telomeric DNA isolated from TRF2-depleted MEFs (Fig5A) showed a significant reduction in the 3′ overhang signal compared to control cells (Fig5B and C). Importantly, CtIP inhibition did not restore the single-stranded DNA signal to the level of GFP shRNA-treated control cells, suggesting that CtIP is not required for 3′ overhang excision.

53BP1 counteracts CtIP- or BLM-mediated resection at intrachromosomal DSB (Bunting *et al*, 2010; Grabarz *et al*, 2013). Furthermore, at telomeres uncapped through TRF2 deletion, 53BP1 prevents extension of the 3′ overhang (Dimitrova *et al*, 2008). Consistent with this, we found that 53BP1 co-depletion with TRF2 reduced single-stranded telomeric DNA to control levels (Fig5B and C). As CtIP is known to stimulate resection, we examined whether CtIP plays a role in telomere processing in the context of 53BP1 inactivation. Depletion of CtIP together with 53BP1 and TRF2 did not affect single-stranded DNA formation, suggesting that CtIP is not required for the extensive resection induced by loss of 53BP1 at telomeres uncapped by TRF2 removal. This is contrary to telomeres lacking the entire shelterin complex, where 53BP1 abrogation leads to extensive resection, partly dependent on CtIP (Sfeir & de Lange, 2012). Importantly, in our experimental set-up, CtIP remained critical for resection at intra-chromosomal breaks induced by ionizing radiation. This is illustrated by impaired RAD51 focus formation upon CtIP depletion, both in normal cells and in cells lacking 53BP1 (Supplementary Fig S4). Moreover, we monitored the effect of BRCA1 on the levels of telomere fusions in 53BP1/TRF2 shRNA-treated cells. BRCA1 antagonizes 53BP1 at intra-chromosomal DSBs, and thus, we examined the possibility that BRCA1 co-inactivation could restore the decreased fusion frequency caused by 53BP1 abrogation at uncapped telomeres (Supplementary Fig S5). However, Cre-induced *Brca1* deletion did not alter telomere fusion levels in 53BP1-deficient cells, suggesting that the extended telomere overhang generated in the absence of 53BP1 (Fig5B) persists when *Brca1* is deleted, similarly to CtIP inactivation.

### EXO1 is also required for end-joining of uncapped telomeres

**Figure 6:**
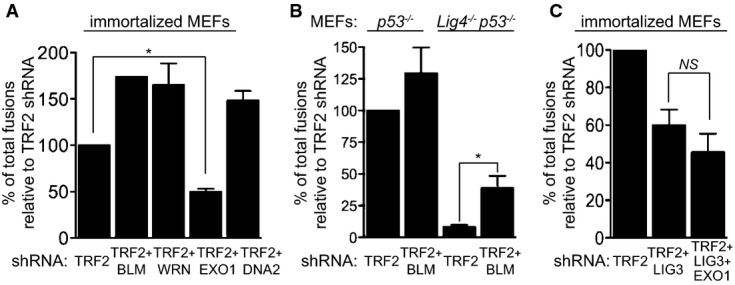
EXO1, but not BLM, WRN or DNA2, promotes LIG3-dependent end-joining of telomeres uncapped by TRF2 inactivation
MEFs of the indicated genotypes were infected with retroviruses expressing the indicated shRNAs, followed by selection with puromycin for 72 h. Mitotic chromosomes isolated 48 h later were fixed and stained with a Cy3-conjugated (CCCTAA)_3_-PNA probe. The frequency of end-to-end chromosome-type fusions is represented as a percentage of fusions observed after TRF2 depletion in each experiment. A minimum of 1,200 chromosomes were scored for each sample. Error bars represent SD of two independent experiments. *P*-values were calculated using an unpaired two-tailed *t-*test. *P ≤ 0.05; NS, *P *> 0.05. Each graph represents a separate set of experiments.

Following initiation of resection by the MRN complex and CtIP, additional factors including EXO1, DNA2, WRN and BLM (Mimitou & Symington, 2009; Huertas, 2010) act to promote further resection. We investigated whether any of these factors are required to process telomeres uncapped through TRF2 removal by inactivating each one of them in TRF2-depleted MEFs. The reduction in expression levels was determined for each of these factors by qPCR or Western blotting (Supplementary Fig S6A and B). Interestingly, only EXO1 inhibition together with TRF2 shRNA caused a significant decrease in the number of telomere fusions when compared to TRF2 depletion alone (Fig6A). This effect is not due to changes in the proliferation rate of these cells (Supplementary Fig S7). In contrast, BLM, WRN and DNA2 inactivation together with TRF2 shRNA led to elevated levels of telomeric fusions (Fig6A), suggesting that these factors act to suppress end-joining at uncapped telomeres. As BLM is known to inhibit A-NHEJ (Grabarz *et al*, 2013), we addressed whether these additional fusions are mediated by C- or A-NHEJ. TRF2 and BLM co-depletion in *Lig4*^*−/−*^ MEFs, monitored by Western blotting and qPCR (Supplementary Fig S8), led to an increase in fusion frequency relative to TRF2 inhibition alone (Fig6B). This indicates that BLM inactivation can de-repress a LIG4-independent pathway for telomere re-joining, likely mediated by LIG3, as previously reported for DSB repair (Grabarz *et al*, 2013). However, we could not directly examine the role of LIG3, because its inactivation together with BLM and TRF2 led to cell cycle arrest (data not shown), which prevented fusion analysis in this genetic background.

In mammalian cells, CtIP interacts with EXO1 in the presence or absence of DNA damage (Eid *et al*, 2010). Although the requirement for CtIP in A-NHEJ is well established (Bennardo *et al*, 2008; Rass *et al*, 2009; Zhang & Jasin, 2011), a similar role for EXO1 has not yet been reported. To address the possibility that EXO1 participates in LIG3-dependent A-NHEJ reactions to mediate fusions of uncapped telomeres, we inactivated LIG3 and EXO1 using shRNAs in cells lacking TRF2. The frequency of end-to-end fusions in TRF2/LIG3-depleted cells (approximately 40.1%; Fig6C) was comparable to that in cells in which EXO1 was additionally depleted (approximately 49.5%) supporting the concept that EXO1 acts in the LIG3-dependent pathway of telomere end-joining.

## Discussion

Telomeric fusions occur between dysfunctional telomeres and pose a clear threat to chromosome integrity. A better understanding of how uncapped telomeres are processed and ligated could unravel the mechanisms that sustain genomic stability in normal cells and could also provide a model to dissect DNA repair in a defined chromosomal context. This study identifies novel activities required for telomere fusions mediated by A-NHEJ and assigns a novel critical function for BRCA1 in this DNA repair pathway.

### Resection-promoting factors BRCA1 and CtIP are required for telomeric end-to-end fusions

**Figure 7:**
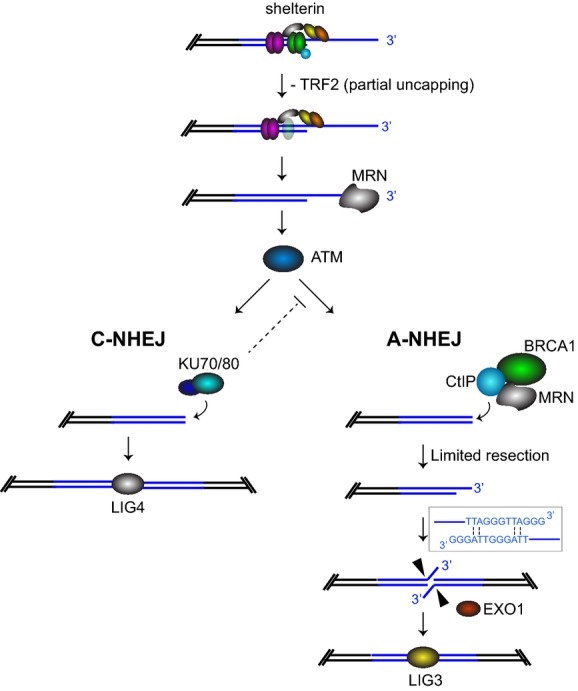
Model for the role of BRCA1-CtIP in A-NHEJ Telomeres uncapped by TRF2 depletion undergo 3′ overhang excision in MRN-dependent manner. Most telomeres are bound by Ku heterodimer and re-joined by C-NHEJ promoted by 53BP1. A subset of the telomeres become substrates for resection reactions, which require MRN, BRCA1 and CtIP. Initiation of resection generates short 3′ telomeric overhangs, whose annealing is facilitated by the homology provided by 2 A-T base pairs per telomeric repeat. The resulting flaps are cleaved by EXO1 and telomeres re-joined through the LIG3-dependent A-NHEJ pathway.

Telomeres uncapped through TRF2 inactivation are processed by nucleolytic activities and become substrates for NHEJ mechanisms that generate end-to-end chromosomal fusions (Fig7). C-NHEJ is the major pathway for dysfunctional telomere re-joining, dependent on functional LIG4 and 53BP1 (Celli & de Lange, 2005; Celli *et al*, 2006; Dimitrova *et al*, 2008). Additionally, uncapped telomeres are fused through A-NHEJ reactions (Sfeir & de Lange, 2012), which are PARP1 and LIG3 dependent (Audebert *et al*, 2004; Simsek *et al*, 2011). In the present work, we demonstrate for the first time that BRCA1 and CtIP are required to process telomeres uncapped through TRF2 abrogation and promote their fusion through the A-NHEJ pathway of DNA repair.

At sites of DNA DSBs, CtIP interacts directly with the MRN complex to trigger end resection with formation of a 3′ single-stranded DNA overhang (Sartori *et al*, 2007; You *et al*, 2009). Following this initiating event, extensive resection occurs in S/G2 to generate long overhangs (100–200 nucleotides), which become substrates for recombinational repair. Alternatively, limited resection (4–6 nucleotides: Ma *et al*, 2003; Guirouilh-Barbat *et al*, 2007) is sufficient to uncover short patches of microhomology that anneal and are processed for end-joining through A-NHEJ reactions. Both MRN and CtIP have been identified as components of the A-NHEJ pathway in mammalian cells (Bennardo *et al*, 2008; Rass *et al*, 2009; Xie *et al*, 2009; Zhang & Jasin, 2011).

At DSB sites, BRCA1 interacts directly with CtIP (Yu *et al*, 1998) and the MRN complex (Chen *et al*, 2008) to promote end resection and repair by homologous recombination. BRCA1 was also shown to increase fidelity of C-NHEJ at I-SceI-induced breaks, mediated by its interaction with Ku80 (Jiang *et al*, 2013). Assembly of distinct BRCA1 complexes with different DNA repair factors may explain this duality. However, the relative abundance or functional interplay between these complexes during physiological cell cycle progression remains unclear. The results presented here support the notion that BRCA1 acts in the A-NHEJ pathway to promote fusion of dysfunctional telomeres. This process is independent of its E3 ubiquitin ligase activity, but requires CtIP and LIG3, both well-established components of A-NHEJ repair. We propose that following TRF2 inactivation and MRN-mediated excision of the 3′ overhang (Deng *et al*, 2009; Dimitrova & de Lange, 2009), chromosome ends elicit ATM activation, which in turn promotes either telomere ligation via C-NHEJ or their resection dependent on BRCA1 and CtIP for A-NHEJ repair (Fig7). Subsequent annealing of the short overhangs generated through resection is facilitated by 2 A-T base pairs per telomeric repeat (Sfeir & de Lange, 2012), which stabilize interactions between chromosome ends and promote their LIG3-dependent re-joining.

CtIP has been previously implicated in the resection of telomeres uncapped by complete removal of shelterin, when 53BP1 is concomitantly inactivated (Sfeir & de Lange, 2012). Under these conditions, shelterin-free ends become prone to extensive resection, which is partially reversed by CtIP inhibition. The data presented here indicate that CtIP plays a different role in processing telomeres uncapped through shRNA-mediated depletion of TRF2. Firstly, loss of CtIP in this context does not rescue extensive telomeric resection occurring when 53BP1 is inactivated (Fig5B and C). This is likely due to the fact that in the absence of TRF2, other shelterin components remain associated with chromosome ends (Takai *et al*, 2010), which limits resection even in the absence of 53BP1. In this context, it would be informative to also examine whether RIF1, a 53BP1 interacting partner (Chapman *et al*, 2013; Escribano-Díaz *et al*, 2013; Feng *et al*, 2013), has the same effect at uncapped telomeres. Secondly, the 3′ single-stranded telomeric DNA is not restored in TRF2-depleted cells (but 53BP1-proficient) when CtIP is concomitantly inactivated (Fig5B and C), although CtIP is functional when damage is introduced intra-chromosomally. This suggests that CtIP-dependent processing occurs at a stage other than overhang excision. Thirdly, CtIP acts in the same telomere re-joining pathway as LIG3, indicative of a role in processing chromosome ends for ligation via A-NHEJ. This most likely occurs through partial resection, which reveals a small number of telomeric repeats sufficient to promote overhang annealing and telomere fusions. However, visualization of such short overhangs of telomeric DNA is not feasible under the experimental conditions used here.

Interestingly, our results show that approximately 50% of the telomeres uncapped by loss of TRF2 require BRCA1 and CtIP for their re-joining through LIG3-mediated reactions. In TRF2-depleted *Ku80*^*−/−*^ MEFs, we find that approximately 64.7% of telomeres remained fused, suggesting that A-NHEJ becomes activated in the absence of Ku80. This is consistent with a previous study in which shelterin removal in *Ku80*^*−/−*^ MEFs led to approximately 60% of telomeres being fused in LIG3- and PARP-1-dependent manner (Sfeir & de Lange, 2012). Paradoxically, however, in both studies only approximately 6–10% of telomeres remain fused in *Lig4*^*−/−*^ MEFs. This could reflect previously reported discrepancies between Ku80 (representative of the Ku/DNA-PKcs/Artemis complex) and XRCC4 (representative of the XRCC4/LIG4 complex) in promoting efficient DSB repair, with the former playing a relatively minor role in enzymatic DNA re-joining (Guirouilh-Barbat *et al*, 2007; Schulte-Uentrop *et al*, 2008). Consistently, *Ku80*^*−/−*^ mice are viable (Zhu *et al*, 1996), whilst *Xrcc4*^*−/−*^ or *Lig4*^*−/−*^ mice die early during embryonic development (Barnes *et al*, 1998; Gao *et al*, 1998). The ability of Ku80 to limit access of the repair factors to broken DNA ends could provide an explanation for these phenotypic differences. Supporting this, *Ku80* knockout restores the viability of *Lig4*^*−/−*^ mice (Karanjawala *et al*, 2002), thus enabling repair of the DSBs accumulated in the absence of LIG4. In the context of uncapped telomeres, Ku80 acts to suppress A-NHEJ, and therefore, the impact of its deletion on fusion formation does not appear to be as substantial as LIG4 inactivation, which drives C-NHEJ repair. Given that the effect of its deletion is so dramatic, it is tempting to speculate that LIG4 acts in concert with LIG3 in telomere re-joining. However, further studies are required to elucidate the interplay between these two ligases in the final stages of DNA repair.

### Multiple DDR factors act at telomeres uncapped through TRF2 depletion

The data presented here demonstrate that a large fraction of end-to-end fusions in TRF2-depleted cells require A-NHEJ, which involves factors including BRCA1, CtIP, EXO1 and LIG3 (Fig7). The MRN complex has also been implicated in the resection step of A-NHEJ at intra-chromosomal DSBs (Rass *et al*, 2009; Xie *et al*, 2009), and it is likely to perform a similar function at uncapped telomeres. However, such a role is difficult to dissect experimentally from the MRN function in telomeric DNA damage signal amplification and overhang excision.

The direct interaction between the MRN complex and CtIP (Sartori *et al*, 2007) is required to initiate 5′–3′ DSB resection (Mimitou & Symington, 2011). Extensive resection is subsequently accomplished through the coordinated actions of EXO1, BLM/WRN helicases and DNA2 endonuclease (Gravel *et al*, 2008; Mimitou & Symington, 2008; Nimonkar *et al*, 2008, 2011; Zhu *et al*, 2008) to generate long recombinogenic 3′ overhangs. Our results indicate that inactivation of either BLM, WRN or DNA2 in the context of TRF2 depletion stimulates end-joining of uncapped telomeres. Abrogation of each of these factors is known to trigger telomere damage (Chu & Hickson, 2009; Lin *et al*, 2013), which may have an additive effect to TRF2 inactivation in fusion formation. Among them, BLM was the only one reported to prevent MRN/CtIP-mediated A-NHEJ during DSB repair (Grabarz *et al*, 2013). Consistent with BLM suppressing A-NHEJ of dysfunctional telomeres, we find that reactivation of a LIG4-independent pathway of telomere joining accounts for the substantial increase in telomere fusions observed in BLM- and TRF2-deficient cells (Fig6B).

Interestingly, EXO1 abrogation decreased the number of fusions induced by loss of TRF2 to levels similar to those triggered by BRCA1 and CtIP inactivation. Moreover, LIG3 and EXO1 act in epistatic manner on the re-joining of telomeres lacking TRF2, suggesting that EXO1 promotes A-NHEJ at uncapped telomeres. In human cells, the interplay between CtIP and EXO1 in resection is currently unclear. A direct interaction between human EXO1 and CtIP has been reported (Eid *et al*, 2010), and CtIP acts to suppress EXO1-dependent long-range resection. *In vitro* reconstitution experiments indicated that initial processing of DNA ends by MRN and CtIP stimulates EXO1-dependent resection (Nicolette *et al*, 2010). However, the latter still occurs in cells depleted of MRN or CtIP, albeit with a slower kinetics (Tomimatsu *et al*, 2012). Nevertheless, extensive resection is not a pre-requisite for repair through A-NHEJ; therefore, it is unlikely that EXO1 acts in concert with CtIP at uncapped telomeres. This notion is also supported by recent studies in *Caenorhabditis elegans* where CtIP and EXO1 act in parallel pathways of DSB repair during meiosis (Lemmens *et al*, 2013). Importantly, EXO1 is a 5′→3′ exonuclease, as well as a structure-specific flap endonuclease (Lee & Wilson, 1999). Thus, a more plausible scenario is that EXO1 acts in A-NHEJ to promote endonucleolytic cleavage of the 3′ flaps generated by partial resection (Fig7), a pre-requisite for the LIG3-mediated ligation step of the reaction.

It is conceivable that multiple pathways become engaged at telomeres that have lost the TRF2-dependent capping structures. De-protected telomeres can be bound by 53BP1 to further engage C-NHEJ, or they can recruit factors promoting resection. Here, we identified BRCA1, CtIP and EXO1 as novel activities required for nucleolytic processing of telomeres lacking TRF2. End-joining of uncapped telomeres occurs primarily in G1, a stage of the cell cycle where extensive resection is suppressed. Short overhangs can be generated by limited resection and channelled into A-NHEJ, the only pathway proficient in re-joining partially resected ends. In addition, BRCA1/CtIP can also promote A-NHEJ during S/G2 through inhibition of RIF1, the 53BP1 interacting partner (Chapman *et al*, 2013; Escribano-Díaz *et al*, 2013; Feng *et al*, 2013). Whether RIF1 blocks A-NHEJ and whether this effect can be counteracted by BRCA1/CtIP remain to be determined. Our study suggests a previously unanticipated importance of A-NHEJ in generating telomere end-to-end fusions, which highlights the complexity of the end-processing reactions. Unravelling the mechanisms of uncapped telomere processing will help clarify how cells preserve telomere integrity and their tumour suppressor potential during normal cellular proliferation.

## Materials and Methods

### Cell lines, culture conditions and treatments

Primary MEFs were isolated from day 13.5 embryos as previously described (Blasco *et al*, 1997) and cultivated in DMEM (Life Technologies) supplemented with antibiotics and 10% foetal bovine serum (Life Technologies) in a low oxygen (3%) incubator. *Brca1*^*F/−*^ MEFs (previously referred to as *Brca1*^*SCo/−*^; Bouwman *et al*, 2010), in which *Brca1* exons 5 and 6 are floxed and flanked by the puromycin resistance gene, were immortalized by overexpression of SV40 large T (LT) antigen or p53 shRNA. In addition, *Brca1*^*F/C61G*^ MEFs (Drost *et al*, 2011) were immortalized by TBX2 overexpression and *Ku80*^*−/−*^ MEFs (a gift of Dr. Maria Blasco, CNIO, Madrid) were LT-immortalized. *Lig4*^*−/−*^*p53*^*−/−*^ MEFs were a gift of Dr. Penny A. Jeggo, University of Sussex. MEFs were arrested in mitosis by addition of 0.2 μg/ml KaryoMAX^**®**^ colcemid (Life Technologies) to the media, followed by 6- to 12-h incubation at 37°C.

### MEF retroviral transduction

For retroviral transduction of cultured MEFs (Palmero & Serrano, 2001), HEK293T packaging cells were grown to 70% confluency and transfected with pCL-Eco helper vector together with either pBabe alone or pBabe plus retroviral vector encoding ‘Hit-and-run’ Cre recombinase (Silver & Livingston, 2001) using a standard calcium phosphate protocol. For shRNA transfection, pCL-Eco helper vector was co-transfected along with retroviral constructs in pRetroSuper encoding control GFP shRNA (GCT GAC CCT GAA GTT CAT CTT; Tarsounas *et al*, 2004), TRF2 shRNA (GAA CAG CTG TGA TGA TTA A; Deng *et al*, 2009), BRCA1 shRNA (GCC TCA CTT TAA CTG ACG CAA T, used as a 1:1 mix with lentiviral-expressed BRCA1 shRNA, see sequence below), CtIP shRNA (in pMX-pie-IRES-GFP: GCT CTC TAT GTA CAA ATG AAT TA; Bunting *et al*, 2010), 53BP1 shRNA (GCT ATT GTG GAG ATT GTG TTT; Bouwman *et al*, 2010), NBS1 shRNA (TTT GAC TCA AAC TGG TTAC; a gift of Dr. J. Jacobs, NKI, Amsterdam), MRE11 shRNAs (a 1:1 mix of GCT TGT AAG AAC TTG GCT AAA and GCG CAT TAA AGG GAG AAA GAT), EXO1 shRNAs (a 1:1 mix of GGG TCA AGC CGA TTC TCA TAT TT and GTC AAG CCG ATT CTC ATA TTT), DNA2 shRNAs (a 1:1 mix of GGG AGA TCA GAG GAC TAT TAC and ATG GCA GGT GAC AGG ATT ATT), BLM shRNAs (a 1:1 mix of GCC AGG TTA TCT GTG CGA CAA T and CGA AGG AAA CTC ACG TCA ATA) and WRN shRNAs (a 1:1 mix of GCA AGG CAG AAA CAC GCT AAT and GTC GAG CCA TAG AGT CTT TAA T). The medium was replaced 24 h after transfection. Recipient MEFs were plated and infected 24 h later with the retroviral supernatants produced by the HEK293T cells. Infections were repeated after 24 and 32 h. The following day, cells were washed and incubated in fresh medium containing 2–3 μg/ml puromycin for 48 h. MEFs were either processed for Western blot analysis or for metaphase chromosome preparation as described below.

### MEF lentiviral transduction

For lentiviral transduction of cultured MEFs (Dull *et al*, 1998), HEK293T packaging cells were grown to 70% confluency and transfected with Gag-Pol, Res-Rev and VSV-G packaging vectors together with shRNA-containing pLKO.1 constructs. The hairpins used were as follows: control luciferase shRNA (CGC TGA GTA CTT CGA AAT GTC; Deng *et al*, 2009), LIG3 shRNA (CCA GAC TTC AAA CGT CTC AAA), BRCA1 shRNA (Sigma; TRCN306222; GTG CTT CCA CAC CCT ACT TAC, used as a 1:1 mix with retroviral-expressed BRCA1 shRNA) and PARP1 (Sigma; TRCN325059; GCC CTT GGA AAC ATG TAT GAA). The medium was replaced 24 h after transfection. Recipient MEFs were plated and infected twice at 24-h intervals with the lentiviral supernatants produced by the HEK293T cells. Twenty-four hours after the last infection, cells were washed and incubated in fresh medium containing 2–3 μg/ml puromycin for 3–5 days. MEFs were processed as described for the retroviral transduction.

### Real-time quantitative PCR (qPCR)

Real-time RT–PCR using the PowerSYBR® Green Cells-to-CT™ kit (Ambion) was performed after puromycin selection in order to determine *Exo1, Dna2* and *Blm* mRNA levels. Reverse transcriptase reactions were performed in a Veriti® 96-Well Thermal Cycler (Applied Biosystems, Life Technologies Corporation). PCRs were performed using a StepOnePlus™ Real-Time PCR System (Applied Biosystems, Life Technologies Corporation). The primers used to amplify mouse transcripts were as follows: *Exo1* mRNA: ACC AGC TCG TGT TCG ACC CCA and CAC CGA CGT ACT GCC CAG CG, *Dna2* mRNA: ACC ACC ATC TGT GCC CTG GTG A and AAC GGC GGA GTG CGT GTA GC and *Blm* mRNA: TGT CGG CGC GCG GAG TTT and ACA GCA GCC ATG ATC CTC ACT CA. Primers for mouse β-actin (GCT CTG GCT CCT AGC ACC AT and CCA CCG ATC CAC ACA GAG TAC) were used as an endogenous control. All qPCR reactions were performed in triplicate.

### FACS analysis

Cells harvested by trypsinization were washed in cold PBS and fixed in ice-cold ethanol (70%) for 16 h at −20°C. Fixed cells were washed in PBS and incubated with 5 μg/ml propidium iodide and 0.25 mg/ml RNase I (Sigma-Aldrich) in PBS. A minimum of 10,000 cells were analysed by flow cytometry (Becton Dickinson), and resulting data were processed using CellQuest (Becton Dickinson).

### Cell proliferation assay

Twenty thousand MEFs were seeded in each well of a 6-well plate, and cell numbers were determined 24, 48 and 96 h after plating, following treatment with resazurin (10 μg/ml) for 2 h. Fluorescence was measured at 590 nm using a micro-titre plate reader (EnVision; Perkin Elmer).

### Immunoblotting

Cells harvested by trypsinization were washed with cold PBS, re-suspended in SDS–PAGE loading buffer (2 × 10^7^ cells/ml) and sonicated. Equal amounts of protein (50–100 μg) were analysed by gel electrophoresis and Western blotting. NuPAGE-Novex 10% Bis-Tris, NuPAGE-Novex 3–8% Tris-Acetate gels (Life Technologies) and Clearpage 10% SDS gels (C.B.S.-Scientific) were run according to manufacturer's instructions. Anti-α-tubulin, GAPDH or SMC1 antibodies were used as loading controls.

### Detection and quantification of single-stranded telomeric DNA using pulsed-field gel electrophoresis

Approximately 1 × 10^6^ MEFs were harvested by trypsinization and stored as frozen pellets. Telomeric overhang analysis was performed as previously described (Sfeir & de Lange, 2012). Briefly, cells were re-suspended in PBS, mixed with 2% agarose (1:1 ratio) and casted in a plug mould. The plugs were then digested overnight at 55°C in proteinase K digestion buffer (10 mM Tris–HCl pH 8.0, 250 mM EDTA, 0.2% sodium deoxycholate and 1% sodium lauryl sarcosine). After washes with TE, plugs were digested with 60 U MboI and 60 U AluI overnight at 37°C. The cleaved DNA was separated on a 1% agarose/0.5× TBE gel, which was then dried at room temperature and hybridized overnight at 50°C with γ-^32^P-ATP end-labelled (AACCCT)_4_ probe in Church mix (0.5 M sodium phosphate, pH 7.2, 1 mM EDTA, 0.7% SDS, 0.1% BSA). Following three washes at 50°C with 4× SSC (30 min each) and one wash with 4× SSC/0.1% SDS (30 min), the dried gel was exposed to a Phosphor Imager screen. Upon acquisition of the single-stranded telomere signal, the gel was denatured with 0.5 M NaOH/1.5 M NaCl for 30 min, neutralized with two 30-min washes in 0.5 M Tris–HCl pH 7.5/3 M NaCl, pre-hybridized in Church mix at 55°C (30 min) and hybridized overnight at 55°C with the γ-^32^P-ATP end-labelled (AACCCT)_4_ probe. The denatured gel was washed as above and exposed to capture the total telomere signal. ImageQuant software was used to quantify both the single-stranded and total telomeric DNA.

### Preparation of metaphase spreads and telomere FISH

Colcemid-treated cells collected by mitotic shake-off were swollen in hypotonic buffer (0.03 M sodium citrate) at 37°C for 25 min and re-suspended in freshly prepared 3:1 mix of methanol:glacial acetic acid. Fixed chromosomes were dropped onto slides pre-soaked in 45% acetic acid and left to dry overnight. For telomeric FISH (Thanasoula *et al*, 2010), the telomeric probe mix containing 10 mM Tris pH 7.5, 2.175 mM MgCl_2_, 0.08 mM citric acid, 7.2 mM Na_2_HPO_4_ pH 7.0, 70% deionized formamide (Chemicon Int.), 0.5 μg/ml Cy3-conjugated PNA (CCCTAA)_3_ telomeric probe (Applied Biosystems) and 0.25% blocking reagent [100 mM maleic acid and 150 mM NaCl pH 7.5 (Roche) in dH_2_O] was dropped onto each slide and sealed with a coverslip. Following denaturation on a hot plate for 3 min at 80°C, the slides were incubated at room temperature for 1.5 h in a dark humidified chamber. After washing twice in formamide [70% formamide (Fluka), 10 mM Tris, 0.1% BSA (Fluka)], three times in PBS, the slides were left to dry at room temperature. Slides were then mounted using Vectashield with 1 μg/ml 4,6-diamidino-2-phenylindole (DAPI; Vector Laboratories). Mitotic chromosomes were viewed with a Leica DMI6000B inverted microscope and fluorescence imaging workstation equipped with a HCX PL APO 100×/1.4–0.7 oil objective. Images were acquired using a Leica DFC350 FX R2 digital camera and LAS-AF software (Leica). Brightness levels and contrast adjustments were applied to the whole image using Photoshop CS3 (Adobe).

### Immunofluorescence-combined FISH (IF-FISH)

MEFs grown in monolayer were fixed in 4% paraformaldehyde with 0.1–0.5% Triton X-100 in PBS and permeabilized with 0.5% Triton X-100 in PBS. For immunofluorescence staining (Tarsounas *et al*, 2004), cells were incubated in antibody dilution buffer (1% goat serum, 0.3% BSA, 0.005% Triton X-100 in PBS) for 20 min and then in primary antibody overnight at room temperature. Following three washes in antibody dilution buffer supplemented with 0.005% Triton X-100, Alexa 488-conjugated secondary antibody was added for 1 h. Samples were then washed and fixed again in 4% paraformaldehyde in PBS. Following three washes in PBS, slides briefly dried at room temperature were treated as described above for telomere FISH and fluorescence signals were quantified with ImageJ 1.43k (National Institutes of Health, USA).

### Telomere chromosome orientation FISH (CO-FISH)

Cells plated at 50–60% confluency were treated for 20 h with bromodeoxyuridine (BrdU) either alone, or in combination with bromodeoxycytidine (BrdC) in 3:1 ratio, to a final concentration of 10 μM. To obtain metaphases, 0.2 μg/ml colcemid was added to the cells 4–6 h before collection. Mitotic chromosomes were fixed and spread onto slides as described for telomeric FISH and then processed for CO-FISH as previously described (Bailey *et al*, 2001). After washing in PBS for 10 min, the slides were treated with 0.5 mg/ml RNase A (Sigma-Aldrich) in PBS for 10 min at 37°C in a wet chamber. Following washes in PBS and 2× saline-sodium citrate (SSC), the DNA was stained with 0.5 μg/ml Hoechst 33258 (Sigma-Aldrich) in 2× SSC for 15 min in the dark. In order to introduce DNA breaks, slides were abundantly washed with water and then exposed to ultraviolet (UV; 365 nm) light for 25 min. Following PBS washes, the DNA was digested with 3 U/μl of Exonuclease III (Promega). The slides were washed again with PBS and then dehydrated with sequential washes of 70, 90 and 100% ethanol. When dried, the first telomeric probe mix (same as described for telomere FISH) containing 0.5 μg/ml Cy3-conjugated PNA (TTAGGG)_3_ telomeric probe (Applied Biosystems) was added to the slides. DNA was denatured on a hot plate at 80°C for 3 min. Then slides were incubated for 1.5 h in the dark at room temperature and washed twice with formamide wash, as described for telomere FISH. Next, the slides were washed with PBS, dehydrated with ethanol and after air-drying, were incubated with the second telomeric probe mix containing 0.5 μg/ml FITC-conjugated PNA (CCCTAA)_3_ telomeric probe (Applied Biosystems) for 1.5 h in the dark at room temperature. Following washes with formamide wash and PBS and dehydration with ethanol, slides were dried and mounted using Vectashield with DAPI (Vector Laboratories). The images were acquired using the same system as described for IF-FISH.

### Antibodies

The following antibodies were used for immunoblotting: rabbit polyclonal antisera raised against SMC1 (BL308; Bethyl Laboratories), BRCA1 (Bouwman *et al*, 2010), 53BP1 (NB100-304; Novus Biologicals), PARP1 (46D11; Cell Signaling) and human histone H3 (a gift from Dr. Alain Verreault, University of Montreal); mouse monoclonal antibodies raised against TRF2 (NB100-57130; Novus Biologicals), phospho-ATM Ser1981 (Cell Signaling), MRE11 (NB100-142; Novus Biologicals), CHK2/Cds1 (clone 7, Upstate), CtIP (a gift from Dr. Richard Baer, Columbia University), NBS1 (ab49958; Abcam), WRN (a gift from Dr. Thomas Helleday, Karolinska Institute), GAPDH (NB600-502; Novus Biologicals), LIG3 (611876; BD Transduction) and α-tubulin (Cancer Research UK Monoclonal Antibody Service). In addition, rabbit polyclonal antibodies raised against RAD51 (FBE2; Clare Hall Laboratories) and 53BP1 (NB100-304; Novus Biologicals) as well as mouse monoclonal against phosphorylated histone H2AX-Ser139 (JBW301; Upstate) were used for immunofluorescence detection.

## Supporting Information

Supplementary Figures

Review Process File
