# Supplementary material for: BRCA1 and CtIP promote alternative non-homologous end-joining at uncapped telomeres
Source: EMBO J. 2015 Jan 12;34(3):410–24. doi: 10.15252/embj.201488947 (PMC4339125; doi:10.15252/embj.201488947)
Supplement: Supplementary file 1 [file embj0034-0410-sd1.pdf]

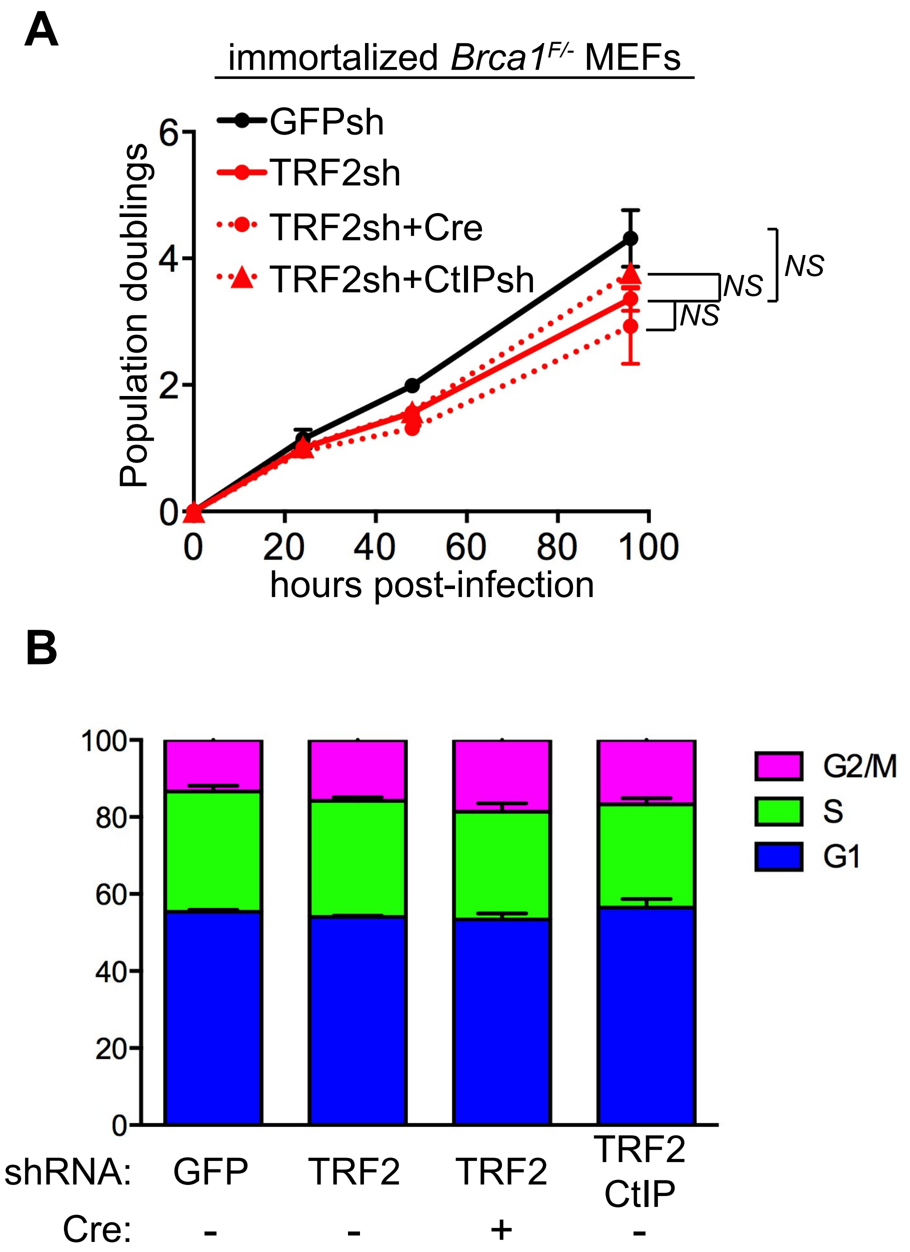

Figure S1. Proliferation and cell cycle progression in MEFs lacking BRCA1, TRF2 and/or CtIP. Immortalized *Brca1*$^{F/-}$ MEFs were infected with retroviruses expressing the indicated shRNAs and/or Cre recombinase, followed by selection with puromycin for 72 hours. (A) Cells were re-plated for cell proliferation assays. Error bars represent SD of three independent experiments. *P* values were calculated using an unpaired two-tailed *t*-test. *NS*, $P>0.05$. (B) Cells treated as in (A) were processed for FACS analyses of DNA content.

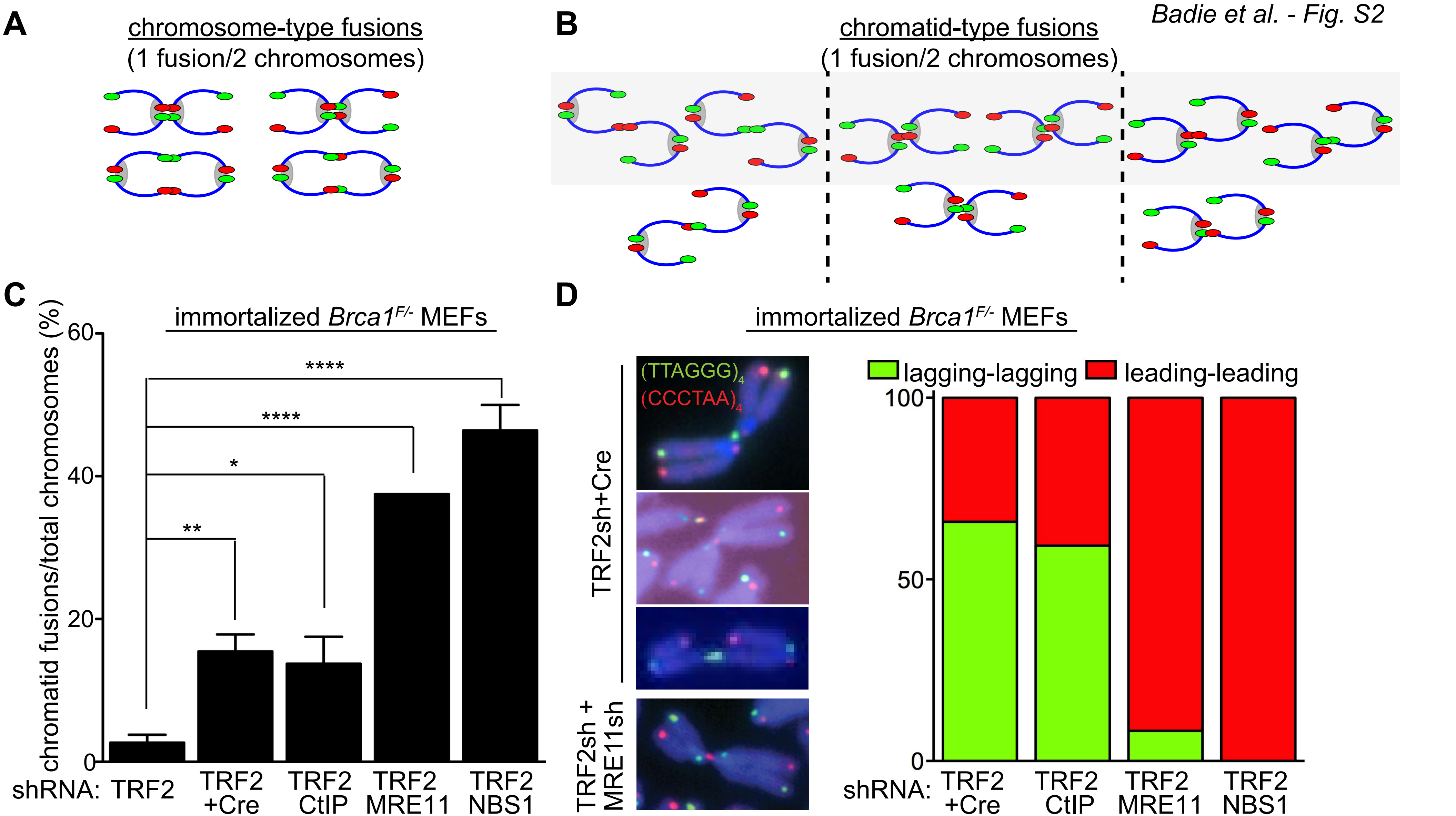

Figure S2. Impact of BRCA1, CtIP, NBS1 or MRE11 inactivation on chromatid-type fusions. Schematic diagram of the method for calculating the frequency of (A) chromosome- and (B) chromatid-type telomeric fusions detected using CO-FISH on mouse mitotic chromosomes spreads. Centromeres are shown in grey. (C) Immortalized *Brca1*$^{F/-}$ MEFs were infected with retroviruses expressing the indicated shRNAs and/or Cre recombinase, followed by selection with puromycin for 72 hours. Cells were arrested in mitosis with colcemid and mitotic chromosomes were processed for telomeric CO-FISH analysis. Metaphase chromosome spreads were stained with a Cy3-conjugated leading strand telomeric PNA probe (red) and an FITC-conjugated lagging strand telomeric PNA probe (green). DNA was counter stained with DAPI (blue). The frequency of chromosome ends engaged in chromatid-type telomeric fusions (illustrated in B) was quantified as a percentage of total number of chromosomes. A minimum of 2000 chromosome ends was scored for each treatment. Error bars represent SD of at least two independent experiments. *P* values were calculated using an unpaired two-tailed *t*-test. *, $P \leq 0.05$; **, $P \leq 0.01$; ****, $P \leq 0.0001$. (D) Quantification of the relative frequency of chromosome ends engaged in lagging-lagging and leading-leading chromatid-type telomeric fusions (grey box in B) observed in cells treated as in (C). A minimum of 2000 chromosome ends was scored for each treatment. Examples of chromatid-type telomeric fusions are shown on the left panels.

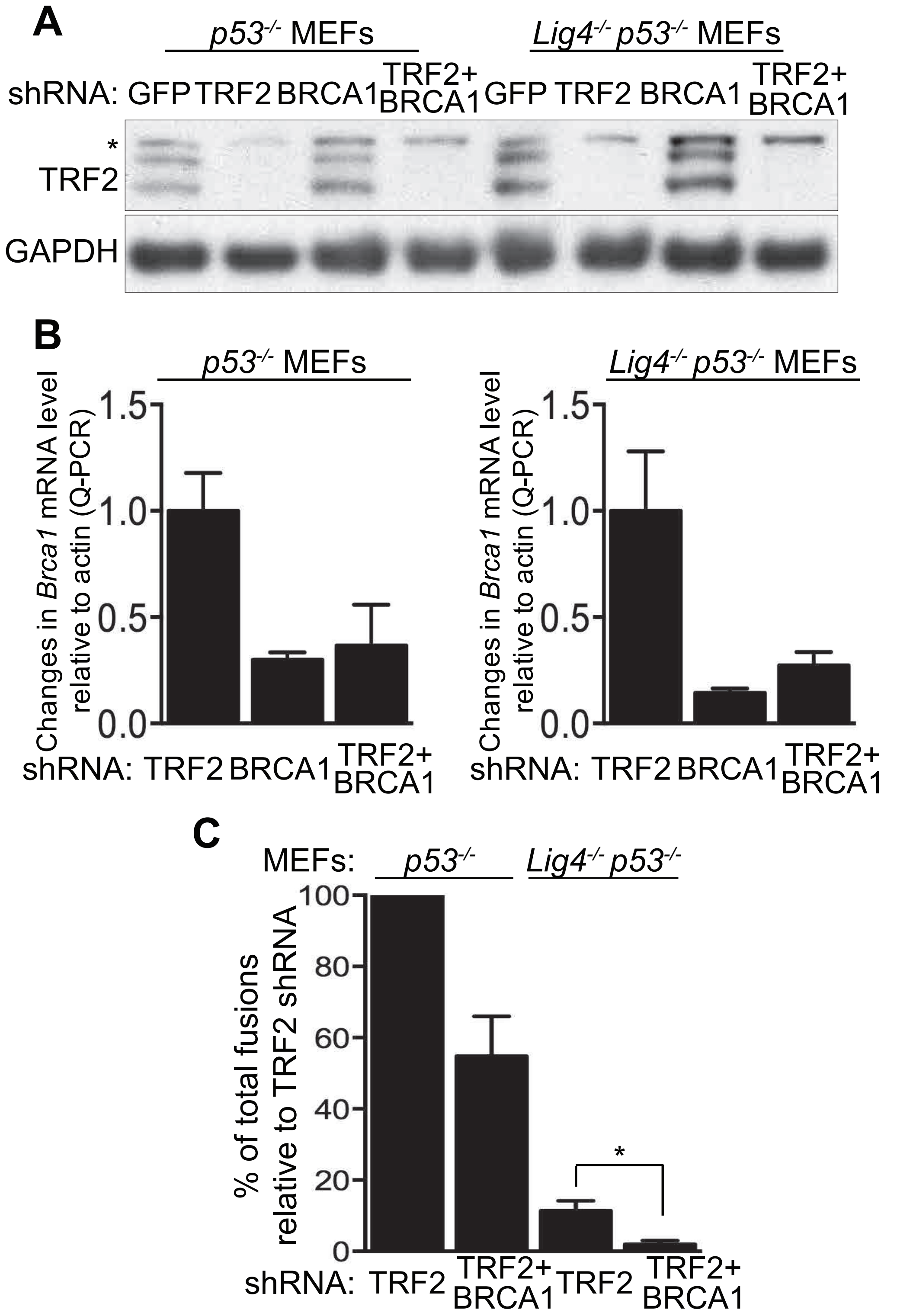

Figure S3. (A) Immortalized *p53*$^{-/-}$ and *p53*$^{-/-}$*Lig4*$^{-/-}$ MEFs were infected with a retrovirus expressing TRF2 shRNAs and/or a 1:1 mix of a retrovirus:lentivirus expressing BRCA1 shRNAs, followed by selection with puromycin for 72 hours. Cell extracts were prepared 48 hours later and analysed by Western blotting as indicated. GAPDH was used as a loading control. *, non-specific band. (B) Efficiency of BRCA1 shRNA-mediated depletion in MEFs treated as in (A). mRNA was isolated 48 hours after selection and the level of *Brca1* transcript was determined using quantitative PCR (Q-PCR). (C) Quantification of the frequency of end-to-end chromosome-type fusions in cells treated as in (A) represented as a percentage of fusions observed after TRF2 depletion. A minimum of 1500 chromosomes was scored for each sample. Error bars represent SD of two independent experiments. The *P* value was calculated using an unpaired two-tailed *t*-test. *, $P \leq 0.05$.

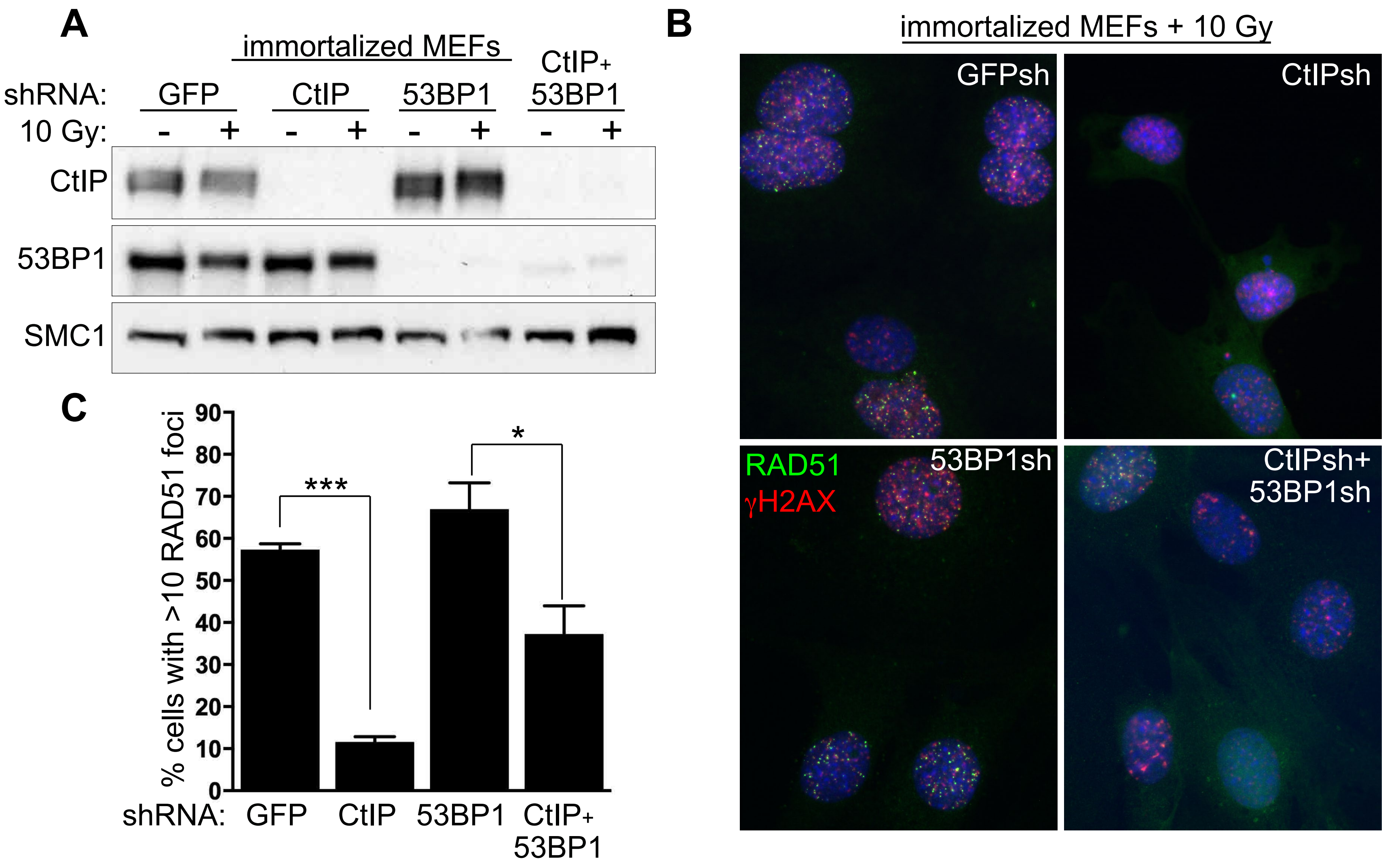

Figure S4. (A) Immortalized MEFs were infected with retroviruses expressing the indicated shRNAs, followed by selection with puromycin for 72 hours. Cells were exposed to 10 Gy of ionizing radiation 24 hours later and allowed to recover for 2 hours. Cell extracts were prepared and analysed by Western blotting as indicated. SMC1 was used as a loading control. (B) Cells treated as in (A) were fixed and stained with antibodies against RAD51 and γH2AX. (C) Quantification of the frequency of RAD51 foci in cells treated as in (A). A minimum of 200 cells were scored for each sample. Error bars represent SD of three independent experiments. The *P* value was calculated using an unpaired two-tailed *t*-test. *, $P \leq 0.05$; ***, $P \leq 0.001$.

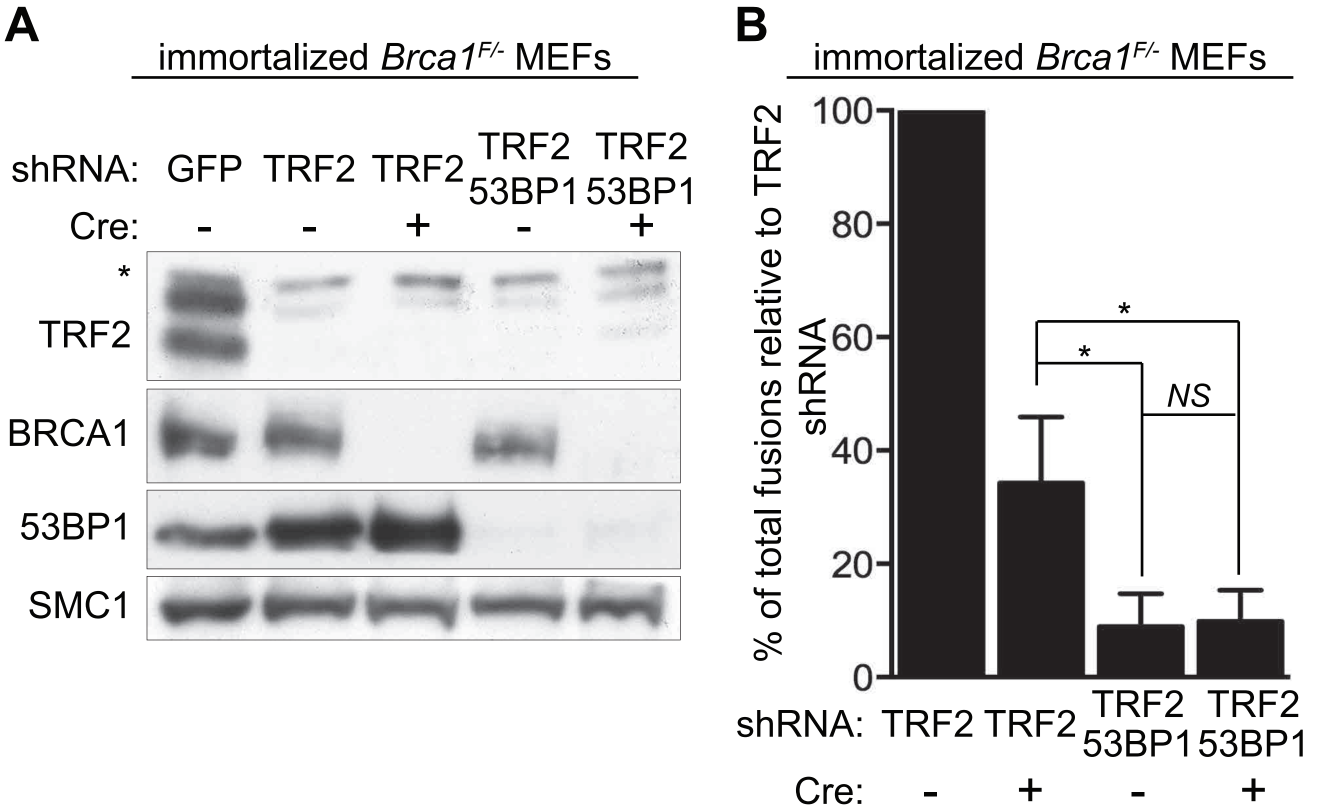

Figure S5. *Brca1* deletion cannot reverse suppression of telomeric fusions induced by 53BP1 shRNA. (A) Immortalized *Brca1*$^{F/-}$ MEFs were infected with retroviruses expressing the indicated shRNAs and/or Cre recombinase, followed by selection with puromycin for 72 hours. Cell extracts were prepared 48 hours later and analysed by Western blotting as indicated. SMC1 was used as a loading control. *, non-specific band. (B) Immortalized *Brca1*$^{F/-}$ MEFs were infected with retroviruses as in (A), followed by selection with puromycin for 72 hours. Mitotic chromosomes isolated from cells treated as in (A) 48 hours after selection were fixed and stained with a Cy3-conjugated $(CCCTAA)_3$-PNA probe. The frequency of end-to-end chromosome-type fusions is represented as a percentage of fusions observed after TRF2 depletion. A minimum of 2000 chromosome ends was scored for each sample. Error bars represent SD of two independent experiments. *P* values were calculated using an unpaired *t*-test. *, $P \leq 0.05$; *N*S, $P > 0.05$.

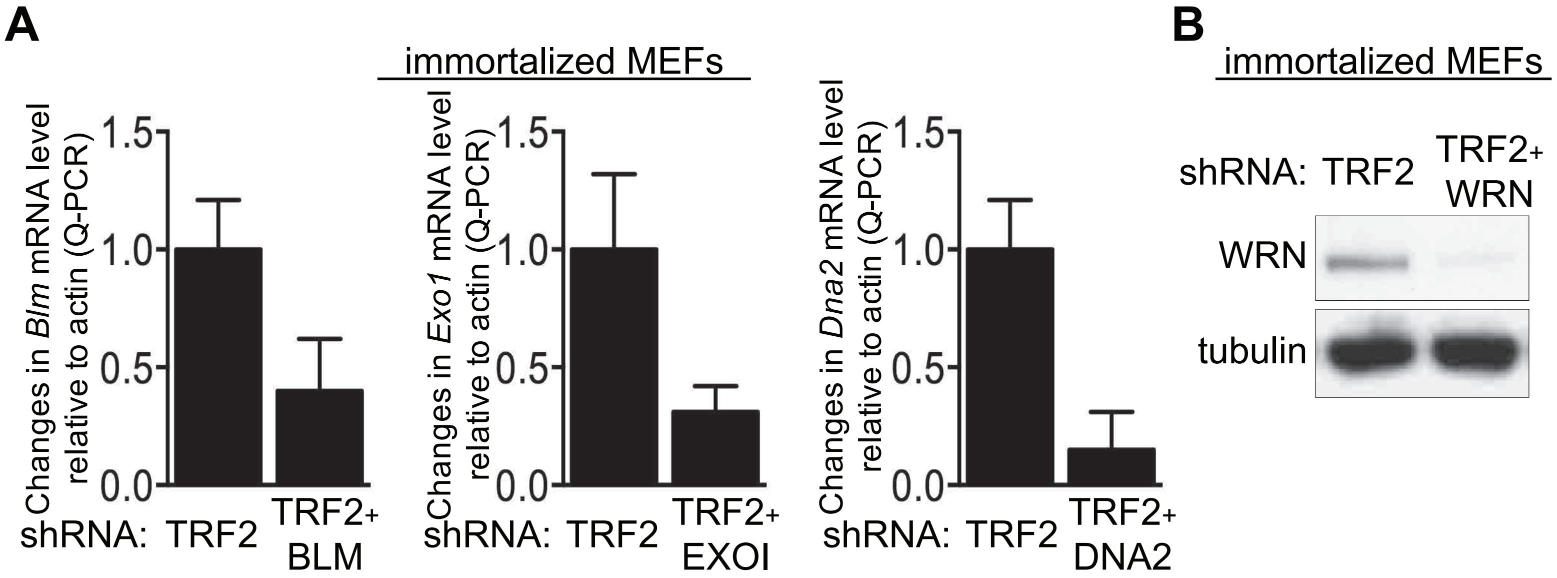

Figure S6. Efficiency of BLM, EXO1, DNA2 and WRN shRNA-mediated depletion in the MEFs analysed in Fig. 5. (A) Immortalized MEFs were infected with retroviruses expressing the indicated shRNAs, followed by selection with puromycin for 72 hours. mRNA was isolated 48 hours later and the levels of indicated transcripts were determined using quantitative PCR (Q-PCR). (B) Immortalized MEFs were infected with retroviruses expressing the indicated shRNAs, followed by selection with puromycin for 72 hours. Cell extracts were prepared 48 hours later and analysed by Western blotting as indicated. Tubulin was used as a loading control.

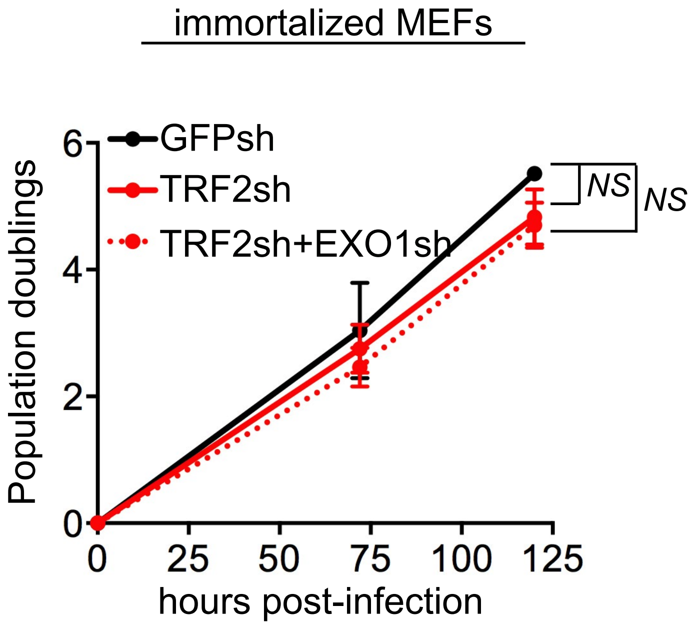

Figure S7. Proliferation and cell cycle progression in MEFs lacking TRF2 and/or EXO1. Immortalized MEFs were infected with retroviruses expressing the indicated shRNAs, followed by selection with puromycin for 72 hours. Cells were re-plated for cell proliferation assays. Error bars represent SD of three independent experiments. *P* values were calculated using an unpaired two-tailed t-test. *N*S, P>0.05

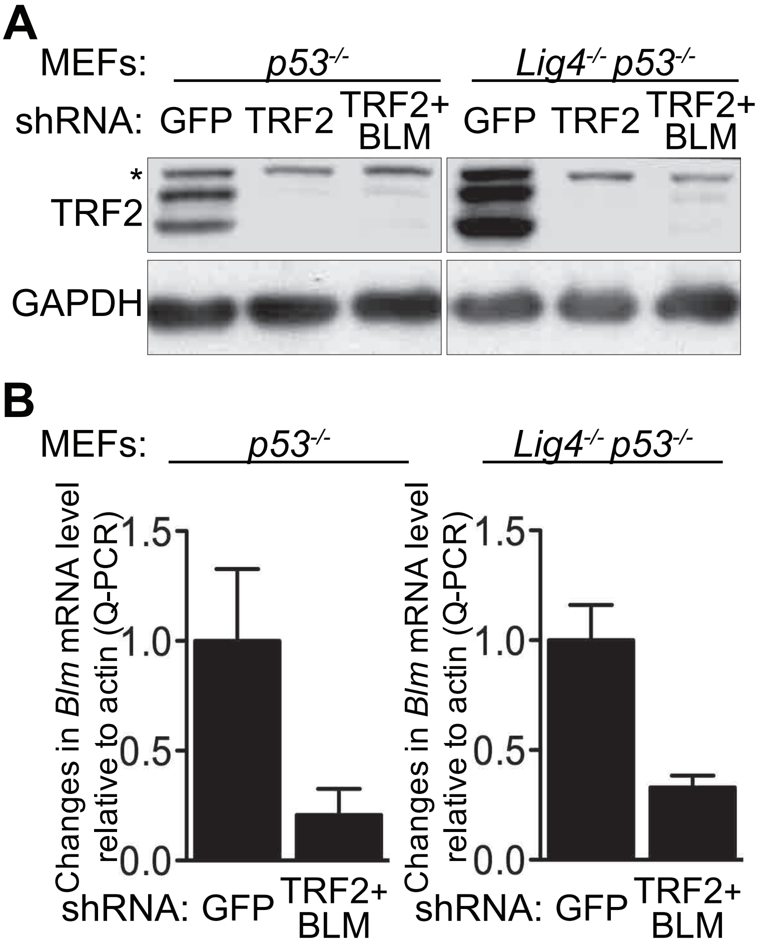

Figure S8. (A) Immortalized *p53*$^{-/-}$ and *p53*$^{-/-}$*Lig4*$^{-/-}$ MEFs were infected with retroviruses expressing the indicated shRNAs, followed by selection with puromycin for 72 hours. Cell extracts were prepared 48 hours later and analysed by Western blotting as indicated. GAPDH was used as a loading control. *, non-specific band. (B) Efficiency of BLM shRNA-mediated depletion in MEFs of the indicated genotypes treated as in (A). mRNA was isolated 48 hours after selection and the level of *Blm* transcript was determined using quantitative PCR (Q-PCR).
