# Supplementary material for: BRCA1 and CtIP promote alternative non-homologous end-joining at uncapped telomeres
Source: EMBO J. 2015 Jan 12;34(3):410–24. doi: 10.15252/embj.201488947 (PMC4339125; doi:10.15252/embj.201488947)
Supplement: Supplementary file 2 [file embj0034-0410-sd2.pdf]

THE
EMBO
JOURNAL

Manuscript EMBO-2014-88947

BRCA1 and CtIP promote alternative non-homologous end-joining at uncapped telomeres

Sophie Badie, Ana Rita Carlos, Cecilia Folio, Keiji Okamoto, Peter Bouwman, Jos Jonkers and Madalena Tarsounas

Corresponding author: Madalena Tarsounas, University of Oxford

Review timeline:

Submission date:	22 May 2014
Editorial Decision:	01 July 2014
Revision received:	29 October 2014
Accepted:	25 November 2014

Editor: Hartmut Vodermaier

Transaction Report:

(Note: With the exception of the correction of typographical or spelling errors that could be a source of ambiguity, letters and reports are not edited. The original formatting of letters and referee reports may not be reflected in this compilation.)

1st Additional correspondence 18 June 2014

Thank you again for submitting your manuscript EMBOJ-2014-88947, "BRCA1 and CtIP promote alternative non-homologous end-joining at uncapped telomeres." We have now received a complete set of reviews from all referees, which you will find enclosed below for your information.

As you will see, the referees' opinions and recommendations are somewhat mixed and equivocal. Overall, there are significant concerns with the actual amount of conceptually novel insight, both in light of precedent from other publications (and potential discrepancies with them) and given that the depth of molecular understanding remains somewhat limited by the mainly genetic/phenotypic nature of the presented analyses. I am therefore afraid that the paper in its present form does not yet appear well suited for publication in The EMBO Journal, but I realize that it may become a more compelling candidate if extended along the lines suggested by the referees.

It would in this situation therefore be very helpful to hear from you what you may be able to do during revision to address the concerns and strengthen the manuscript, before finalizing an editorial decision in this case.
Therefore, I would appreciate if you could carefully consider and discuss the referees' comments with your coworkers, and provide me with a tentative point-by-point response letter, ideally by early next week. I'd also be happy to discuss with you directly (e.g. by phone) on the basis of such a response letter.

Referee #1

In this work, Badie and colleagues show that the tumour suppressor protein BRCA1 and its interacting partner CtIP facilitate joining of uncapped telomeres. TRF2 inactivation in mouse embryonic fibroblasts (MEFs) results in DNA damage signalling at telomeres, removal of the 3' single-stranded telomeric overhang and telomeric end- to-end fusions. The data presented here prove that loss of BRCA1 or CtIP does not affect the activation of the DNA damage response and the degradation of the 3' overhang, however it does reduce the frequency of telomere fusions induced by telomere uncapping. Both classical (C-NHEJ; Ku70/80- and LIG4-dependent) and alternative non-homologous end-joining (A-NHEJ; Ku70/80- and LIG4-independent) contribute to telomere fusions. Interestingly, Badie and colleagues demonstrate that CtIP is synergistic to LIG4 but epistatic to LIG3, indicating that CtIP promotes joining of uncapped telomeres through A-NHEJ. Finally, the authors place EXO1 in the same LIG3-dependent A-NHEJ pathway of telomere end-joining.

Overall, the data presented by Badie and colleagues provide useful information to understand how cells process dysfunctional telomeres and demonstrate a substantial contribution of A-NHEJ in the generation of telomere fusions. Given that there is an increasing interest in understanding A-NHEJ in a variety of contexts, this work will certainly be of interest to the readership of EMBO Journal.

That being said, some of the results presented in this manuscript are in apparent contradiction to previously published data, primarily from the de Lange lab (in particular, the seminal Sfeir et al. 2012 Science). While, I understand that the experimental systems are different, I feel that it is critical that the authors provide additional controls/experiments to firm up the proposed model shown in Figure 6.

Secondly, since it is practically impossible to sequence telomeric fusion events and infer the type of DNA repair events, the manuscript is largely based on genetic and knockdown analyses. With this in mind, I would define A-NHEJ as Ku- and Lig4-independent and end-joining. Since Ku also actively suppresses A-NHEJ, it is easier to study A-NHEJ in contexts of Ku deficiency. The manuscript would have been stronger if some of the analyses had been carried out in Ku-deficient MEFs.

Specific points:

1. As the authors surely know, the effect of BRCA1 on DNA end-resection is somewhat controversial, and so is its requirement for CtIP activation, ubiquitylation and recruitment to DNA damage sites. The model proposed by the authors would be much stronger if a structure-function analysis was carried out to test the requirement of BRCA1 E3 ligase activity in this process or its interaction with CtIP in mediating fusions at uncapped telomeres.

2. Figure 3-C shows that CtIP and LIG4 independently contribute to telomeric fusions, which is a strong argument to suggest that CtIP participates in A-NHEJ. Is this also true for BRCA1?

3. As mentioned above, the authors speculate that the overlapping requirement for LIG3 and LIG4 for telomere end-joining could be explained if the two act together in certain ligation events. If this is the case, then using LIG3 and LIG4 as a means to distinguish between C-NHEJ and A-NHEJ is not appropriate. The authors should test the contribution of BRCA1 and CtIP in conditions where either Ku70/80 or PARP1 are genetically deleted/depleted as a means to support their conclusions.

4. It was previously reported that the increased resection observed in 53BP1-deficient cells is mediated, at least in part, by CtIP not only at dysfunctional telomeres (Sfeir and de Lange, 2012) but also at intra- chromosomal double-strand breaks (Bothmer et al, 2013). However the results presented in Figure 4 contradict this previously published data. Since these results may generate confusion in the field, one additional control I would suggest to carry out would be to test whether depletion of CtIP is required for resection of intra- chromosomal DSBs in conditions of 53BP1 depletion in the cells used for Figure 4. In other words, the authors should see if they can see the role of CtIP in end-resection at non-telomeric sites in the same cells used to determine the function of CtIP in ssDNA generation at dysfunctional telomeres.

5. BRCA1 and CtIP participate in DSB repair pathway choice by suppressing RIF1 in the S and G2 phases of the cycle (work by the Boulton, Durocher and Chen labs). One possibility that has not been considered by the authors is that RIF1 blocks A-NHEJ and that BRCA1 and CtIP can relieve this inhibition. This model might be difficult to address using RNAi-mediated depletions but is certainly worth discussing.

Referee #2

In this manuscript Badie et al., used TRF2 shRNA mediated depletion in BRCA1 null and CtIP shRNA depleted cells to investigate the role of BRCA1 and CtIP in A-NHEJ. Deletion of BRCA1 and CtIP in TRF2 depleted cells leads to significant reduction in chromosome fusions as compared to TRF2 shRNA alone. The investigators further demonstrate that BRCA1, CtIP and EXOIII act in the same pathway as LIG3 to promote A-NHEJ at TRF2 depleted telomeres. Interestingly, both BRCA1 and CtIP do not function in the ATM-dependent telomere damage signaling, or in telomere overhang removal, which are critical for telomere fusions by C-NHEJ. BRCA1 and CtIP abrogation also leads to an increased frequency of chromatid fusions in TRF2-depleted cells, suggesting that these factors are required in postreplicative processing of both lagging and leading telomere strands at uncapped telomeres. Unfortunately, the present manuscript suffers from a lack of novelty. Both the deLange and Chang labs already demonstrate that TRF2 loss promotes A-NHEJ repair at telomeres, and that CTIP is involved in this process. In addition, the role of BRCA1 (Zhong et al., J Biol Chem; 2002)has already been demonstrated to be required in A-NHEJ.

1. The authors should use arrow to indicate total and phosho-Chk2 bands in Figure 1A.
2. Figure 2B suggests that chromosome fusions upon removal of TRF2 in BRAC1 and CtIP depleted cells are reduced to 8.2% and 6.6% as compared to 17.6% in wild type cells. The authors should clarify in the text that residual fusions in BRCA1 and CtIP deleted cells represent C-NHEJ fusions.
3. The authors should reconstitute BRCA1 and CtIP over expressing constructs in TRF2 depleted cells to show the specificity of knockdown.
4. It would be interesting to know which domain of BRCA1 and CtIP are involved in TRF2 depleted A-NHEJ chromosome fusions.
5. BRCA1S988 promotes error-free NHEJ and suppress A-NHEJ (Dueva and Iliakis, Translational Cancer Research; 2013). To gain mechanistic insights into the roles of BRCA1 in A-NHEJ at TRF2 depleted cells, the authors should investigate the function of BRCA1S988 in TRF2 depleted cells.
6. In addition to LIG3, PARP1 also know to promote A-NHEJ. The authors should deplete PARP1 to further strengthen their data suggesting that BRCA1 and CtIP act in the same pathway as LIG3/PARP1 to promote A- NHEJ at TRF2 depleted telomeres.
7. Figure 4. suggests that CtIP is not required for the removal of 3' overhang at uncapped telomeres. Does deletion of BRCA1 leads to a similar phenotype?
8. Figure 5A suggests that BLM, WRN and DNA2 inactivation together with removal of TRF2 lead to elevated level of telomere fusions, suggesting that these factors act to suppress telomere end-joining. BLM/DNA2 is known to prevent A-NHEJ during DSB repair (Karanja et al., Cell Cycle 2014; Grabarz et al., Cell Report 2013). It would be interesting to know whether these fusions are mediated by C-NHEJ or A-NHEJ.
9. μ symbol is messed up in several places in materials and methods.

Referee #3

Telomeres, at the end of the chromosome can be recognized and processed as regular DSB. End-joining of uncapped telomeres can thus generate chromosome fusion leading to genetic instability. Both canonical C- NHEJ and alternative A-NHEJ can join DNA ends and thus uncapped telomeres. In contrast with C-NHEJ, A-NHEJ is initiated by ssDNA resection. Since the resection at DSB, results from the equilibrium 53BP1 versus BRCA1/CtIP, the authors addressed here the question whether this balance also act at uncapped telomeres.
They show that BRCA1/CtIP promotes uncapped telomere fusion, in an epistasis way with LIG3 (which is involved in A-NHEJ).
Accounting for the knowledge on regular DSB repair and the conservation of the processes at telomeres, these data were intuitive, but remained to be established. In addition, the authors show a

novel interesting data on the role of EXO1, which was unexpected from analysis on "regular" DSB repair.
The manuscript is well written and easy to follow and the experiments are well conducted and convincing.

Specific comments:
- The discussion seems a little bit long.
- Page 3, line 5 from bottom. "Citation of KU-independent NHEJ": the following citations might be quoted
for the KU-independent end-joining pathway in mammalian cells: {Feldmann et al., 2000, Nucleic Acids Res,
28, 2585-2596; Guirouilh-Barbat et al., 2004, Mol Cell, 14, 611-623}.
- Page 3, line 2 from bottom, the involvement of PARP1: this paper might be quoted: {Audebert et al.,
2004, J Biol Chem, 279, 55117-55126}. This paper is quoted later in the manuscript.
- Page 4, line 2: "The role of CtIP". This reference might be added: {Bennardo et al., 2008, PLoS Genet, 4,
e1000110} (it is cited later in the manuscript).
- Page 4, {section sign}2, line 7. About MRE11, this paper could be added {Buis et al., 2008, Cell, 135, 85-
96}.
- Page 4, end {section sign}2 (and discussion...). BRCA1 has been shown to interact with KU and to protect
against A-NHEJ {Jiang et al., 2013, J Biol Chem}; this corresponds to an opposite role to CtIP. Could the authors
comment this "duality".
- Page 6/ Could the authors comment (and speculate in discussion) about the differential effects between
MRE11 and CtIP.
- Page 8: Since the ablation of KU and XRCC4 lead to different effects on the efficiency of end-joining
{Guirouilh-Barbat et al., 2007, Proc Natl Acad Sci U S A, 104, 20902-7}, it would be interesting to know
whether it is similar at telomeres (or at least comment).
- Page 8 {section sign}2, CtIP and A-EJ. The following papers (which are quoted later) can also be cited here:
{Bennardo et al., 2008, PLoS Genet, 4, e1000110; Grabarz et al., 2013, Cell Rep, 5, 21-8}.
- Page 9 {section sign}2: 53BP1 counteract resection at DSB: {Grabarz et al., 2013, Cell Rep, 5, 21-8} could
be also quoted.
- Page 10, line 3, {Bennardo et al., 2008, PLoS Genet, 4, e1000110} could also be quoted.
- Page 11{section sign}3, line 6. Ma et. al. is a yeast paper. This has been also described in mammalian cells
{Guirouilh-Barbat et al., 2007, Proc Natl Acad Sci U S A, 104, 20902-7; Guirouilh-Barbat et al., 2004, Mol Cell,
14, 611-623}
- Page 13{section sign}1. Different effects of KU and Lig4 on DSB repair efficiency. This has been described
(and should be quoted) for regular DSB {Guirouilh-Barbat et al., 2007, Proc Natl Acad Sci U S A, 104, 20902-7;
Schulte-Uentrop et al., 2008, Nucleic Acids Res, 36, 2561-9}, consistently with the viability in mice {Karanjawala et al., 2002, DNA Repair (Amst), 1, 1017-26}. These hypotheses should be discussed with regard to the data presented here.

Additional correspondence 26 June 2014

Thank you very much for sending us the insightful reviews of our manuscript entitled "BRCA1 and CtIP promote alternative end joining at uncapped telomeres" and for the opportunity to respond in principle to the referees' concerns. The main experimental additions requested by Referees 1 and 2

were: a) to define the BRCA1 and CtIP domains required for A-NHEJ at uncapped telomeres; b) to repeat the analyses of dysfunctional telomeres in Ku- and/or PARP1-deficient MEFs; c) to provide a control for the role of CtIP in resection of double stranded DNA breaks (DSBs) localized intra-chromosomally and d) to address the function of BLM in suppressing A-NHEJ at uncapped telomeres. We include below a detailed response on how each of the issue raised by these referees would be addressed in a revised version of our manuscript. We hope that with these suggested revisions our manuscript will become a candidate for publication in EMBO J.

I am looking forward to the opportunity to discuss these potential revisions with you over the phone, many thanks for suggesting this.

We, the authors are grateful for the insightful and generally supportive comments of the referees. Point-by-point response:

Referee #1:
In this work, Badie and colleagues show that the tumour suppressor protein BRCA1 and its interacting partner CtIP facilitate joining of uncapped telomeres. TRF2 inactivation in mouse embryonic fibroblasts (MEFs) results in DNA damage signalling at telomeres, removal of the 3' single-stranded telomeric overhang and telomeric end-to-end fusions. The data presented here prove that loss of BRCA1 or CtIP does not affect the activation of the DNA damage response and the degradation of the 3' overhang, however it does reduce the frequency of telomere fusions induced by telomere uncapping. Both classical (C-NHEJ; Ku70/80- and LIG4-dependent) and alternative non-homologous end-joining (A-NHEJ; Ku70/80- and LIG4-independent) contribute to telomere fusions. Interestingly, Badie and colleagues demonstrate that CtIP is synergistic to LIG4 but epistatic to LIG3, indicating that CtIP promotes joining of uncapped telomeres through A-NHEJ. Finally, the authors place EXO1 in the same LIG3-dependent A-NHEJ pathway of telomere end-joining.

Overall, the data presented by Badie and colleagues provide useful information to understand how cells process dysfunctional telomeres and demonstrate a substantial contribution of A-NHEJ in the generation of telomere fusions. Given that there is an increasing interest in understanding A-NHEJ in a variety of contexts, this work will certainly be of interest to the readership of EMBO Journal.

That being said, some of the results presented in this manuscript are in apparent contradiction to previously published data, primarily from the de Lange lab (in particular, the seminal Sfeir et al. 2012 Science). While, I understand that the experimental systems are different, I feel that it is critical that the authors provide additional controls/experiments to firm up the proposed model shown in Figure 6.

Secondly, since it is practically impossible to sequence telomeric fusion events and infer the type of DNA repair events, the manuscript is largely based on genetic and knockdown analyses. With this in mind, I would define A-NHEJ as Ku- and Lig4-independent and end-joining. Since Ku also actively suppresses A-NHEJ, it is easier to study A-NHEJ in contexts of Ku deficiency. The manuscript would have been stronger if some of the analyses had been carried out in Ku-deficient MEFs.

Response: We agree with the referee's view that there are fundamental differences between our system based on TRF2 shRNA-mediated depletion and that used by Sfeir et al. (2012) in which the entire shelterin complex is genetically abrogated and thus removed from the telomeres. We are happy to address apparent discrepancies between the two studies as suggested by the referee. We also agree with the proposed definition of A-NHEJ events as primarily Ku-independent (given the complex interplay between LIG3 and LIG4 at telomeres) and we discuss below how we plan to repeat some of the analyses in Ku-deficient MEFs.

Specific points:
1. As the authors surely know, the effect of BRCA1 on DNA end-resection is somewhat controversial, and so is its requirement for CtIP activation, ubiquitylation and recruitment to DNA damage sites. The model proposed by the authors would be much stronger if a structure-function analysis was carried out to test the requirement of BRCA1 E3 ligase activity in this process or its interaction with CtIP in mediating fusions at uncapped telomeres.

© European Molecular Biology Organization

Response: The referee is right in pointing out that the end-resection roles of BRCA1 and CtIP represent a topic of debate in the field. We propose to address whether the interaction between BRCA1 and CtIP is required at telomeres by expressing the human CtIP variant carrying the S327A mutation in CtIP shRNA-depleted MEFs. Ser327 mutation to Ala is known to abolish the CDK-dependent phosphorylation required for BRCA1/CtIP interaction. To examine the contribution of the BRCA1 E3 ligase activity we will use knock-in MEFs carrying Brca1C61G mutation (available in Jos Jonkers laboratory). This alteration is known to disrupt the RING domain and to abrogate the E3 ubiquitin ligase activity of the BRCA1/BARD1 heterodimer (Drost et al., 2011). We will measure the frequency of end-to-end fusions induced by TRF2 shRNA depletion in cells expressing CtIP S327A or BRCA1C61G relative to the effect of TRF2-depletion in wt or CtIP/ BRCA1-deleted MEFs.

2. Figure 3-C shows that CtIP and LIG4 independently contribute to telomeric fusions, which is a strong argument to suggest that CtIP participates in A-NHEJ. Is this also true for BRCA1?

Response: In order to address the referee's question, TRF2 shRNA-mediated inhibition should be performed in double Lig4-/-Brca1del MEFs. To our knowledge, such a mouse has not yet been generated. As a substitute, we will attempt to establish shRNAs targeting mouse BRCA1 (also so far not reported in the literature) in order to deplete this protein together with TRF2 in Lig4-/- MEFs. In these cells we will then quantify fusion formation.

3. As mentioned above, the authors speculate that the overlapping requirement for LIG3 and LIG4 for telomere end-joining could be explained if the two act together in certain ligation events. If this is the case, then using LIG3 and LIG4 as a means to distinguish between C-NHEJ and A-NHEJ is not appropriate. The authors should test the contribution of BRCA1 and CtIP in conditions where either Ku70/80 or PARP1 are genetically deleted/depleted as a means to support their conclusions.

Response: We will examine the requirement for Ku80 in telomere joining by depleting TRF2, together with CtIP or BRCA1 in Ku80-/- MEFs. We do not have access to Parp1-/- MEFs to perform a similar experiment. Instead, we will attempt to inhibit PARP1 expression using lentivirus-delivered shRNA (as reported by Sfeir et al., 2012) or PARP1 chemical inhibition using olaparib (as in Bunting et al., 2010; Polato et al., 2014), whilst concomitantly inhibiting TRF2 and CtIP/BRCA1 expression with shRNA.

4. It was previously reported that the increased resection observed in 53BP1-deficient cells is mediated, at least in part, by CtIP not only at dysfunctional telomeres (Sfeir and de Lange, 2012) but also at intra-chromosomal double-strand breaks (Bothmer et al, 2013). However the results presented in Figure 4 contradict this previously published data. Since these results may generate confusion in the field, one additional control I would suggest to carry out would be to test whether depletion of CtIP is required for resection of intra-chromosomal DSBs in conditions of 53BP1 depletion in the cells used for Figure 4. In other words, the authors should see if they can see the role of CtIP in end-resection at non-telomeric sites in the same cells used to determine the function of CtIP in ssDNA generation at dysfunctional telomeres.

Response: We agree with the referee that this control will confirm that CtIP acts to resect DSBs localized at intra-chromosomal sites. We will therefore monitor formation of RAD51 foci using immunofluorescence (Polato et al., 2014) and/or phosphorylation of RP-A at Ser4/8 using Western blotting (Bunting et al., 2010) in MEFs lacking 53BP1 and/or CtIP, following exposure to ionizing radiation. These approaches will enable us to determine the genome-wide presence of resected DNA breaks.

5. BRCA1 and CtIP participate in DSB repair pathway choice by suppressing RIF1 in the S and G2 phases of the cycle (work by the Boulton, Durocher and Chen labs). One possibility that has not been considered by the authors is that RIF1 blocks A-NHEJ and that BRCA1 and CtIP can relieve this inhibition. This model might be difficult to address using RNAi-mediated depletions but is certainly worth discussing.

Response: We are grateful for this suggestion. Indeed we do not have access to Rif1-/- MEFs, but will include in the text a discussion of the potential role for RIF1 in telomere fusions.

Referee #2:

In this manuscript Badie et al., used TRF2 shRNA mediated depletion in BRCA1 null and CtIP shRNA depleted cells to investigate the role of BRCA1 and CtIP in A-NHEJ. Deletion of BRCA1 and CtIP in TRF2 depleted cells leads to significant reduction in chromosome fusions as compared to TRF2 shRNA alone. The investigators further demonstrate that BRCA1, CtIP and EXOIII act in the same pathway as LIG3 to promote A-NHEJ at TRF2 depleted telomeres. Interestingly, both BRCA1 and CtIP do not function in the ATM-dependent telomere damage signaling, or in telomere overhang removal, which are critical for telomere fusions by C-NHEJ. BRCA1 and CtIP abrogation also leads to an increased frequency of chromatid fusions in TRF2-depleted cells, suggesting that these factors are required in postreplicative processing of both lagging and leading telomere strands at uncapped telomeres. Unfortunately, the present manuscript suffers from a lack of novelty. Both the de Lange and Chang labs already demonstrate that TRF2 loss promotes A-NHEJ repair at telomeres, and that CTIP is involved in this process. In addition, the role of BRCA1 (Zhong et al., J Biol Chem; 2002)has already been demonstrated to be required in A-NHEJ.

Response: We consider that our work is novel for several reasons: 1) Both the de Lange and Chang *laboratories have shown that CtIP functions in telomere resection and A-NHEJ respectively, specifically in cells lacking 53BP1. 2) In the work of Titia De Lange and colleagues (Sfeir et al., 2012) the entire shelterin complex dissociates from telomeres through concomitant genetic deletion of Trf1 and Trf2. These are fundamental differences from our experimental system, in which TRF2 is removed from telomeres whilst 53BP1 remains intact. 3) No role for BRCA1 in processing dysfunctional telomeres has been reported so far. 4) The work by Zhong et al. (J Biol Chem, 2002) indicates that Brca1-deleted MEFs are defective in microhomology-mediated end joining (MMEJ), assessed at an I-SceI -induced DSB. No epistatic analyses with LIG3 or PARP1 were reported, to definitely place BRCA1 in the A-NHEJ repair pathway. As such, this work can hardly be considered to have assigned an indisputable role for BRCA1 in A-NHEJ.*

1. *The authors should use arrow to indicate total and phosho-Chk2 bands in Figure 1A.*

Response: We will add arrows to label total and phospho-CHK2 band in the WB in Fig. 1A.

2. *Figure 2B suggests that chromosome fusions upon removal of TRF2 in BRAC1 and CtIP depleted cells are reduced to 8.2% and 6.6% as compared to 17.6% in wild type cells. The authors should clarify in the text that residual fusions in BRCA1 and CtIP deleted cells represent C-NHEJ fusions.*

Response: We will include this clarification.

3. *The authors should reconstitute BRCA1 and CtIP over expressing constructs in TRF2 depleted cells to show the specificity of knockdown.*

Response: In our experiments BRCA1 expression was not reduced by shRNA knockdown, but through conditional Brca1 deletion using Cre recombinase. Cells treated with vector alone were used as a control. The CtIP shRNA was previously used in work reported by Sfeir et al. (2012) and Bunting et al. (2010). Restoring CtIP expression to physiological levels in MEFs treated with CtIP and TRF2 shRNA will be performed as described in Point 1, Referee no. 1 above.

4. *It would be interesting to know which domain of BRCA1 and CtIP are involved in TRF2 depleted A-NHEJ chromosome fusions.*

Response: This will be addressed as described in Point 1, Referee no. 1 above.

5. *BRCA1S988 promotes error-free NHEJ and suppress A-NHEJ (Dueva and Iliakis, Translational Cancer Research; 2013). To gain mechanistic insights into the roles of BRCA1 in A-NHEJ at TRF2 depleted cells, the authors should investigate the function of BRCA1S988 in TRF2 depleted cells.*

Response: In human cells, CHK2-dependent phosphorylation of BRCA1 at Ser988 was reported to be required for effective homologous recombination repair (Zhang et al., Mol. Cell Biol. 24, 2004). However, there is no indication in the literature that the same residue is phosphorylated in mouse or

that this phosphorylation variant is functional in mouse cells.

6. In addition to LIG3, PARP1 also know to promote A-NHEJ. The authors should deplete PARP1 to further strengthen their data suggesting that BRCA1 and CtIP act in the same pathway as LIG3/PARP1 to promote A-NHEJ at TRF2 depleted telomeres.

Response: This will be addressed as described in Point 3, Referee no. 1 above.

7. Figure 4. suggests that CtIP is not required for the removal of 3' overhang at uncapped telomeres. Does deletion of BRCA1 leads to a similar phenotype?

Response: We have attempted on several occasions to detect single-stranded DNA in Cre-treated Brca1F/- MEFs, but did not succeed probably due to low numbers of cells recovered after the Cre treatment. However, our unpublished data (below) indicate that the reduction in fusion frequency observed when 53BP1 shRNA is introduced in TRF2-deficient cells is not reversed when Brca1 is concomitantly deleted using Cre treatment. This suggests that the long overhang generated in the absence of 53BP1 persists when Brca1 is deleted, similarly to CtIP inactivation (Fig. 4C).

8. Figure 5A suggests that BLM, WRN and DNA2 inactivation together with removal of TRF2 lead to elevated level of telomere fusions, suggesting that these factors act to suppress telomere end-joining. BLM/DNA2 is known to prevent A-NHEJ during DSB repair (Karanja et al., Cell Cycle 2014; Grabarz et al., Cell Report 2013). It would be interesting to know whether these fusions are mediated by C-NHEJ or A-NHEJ.

Response: This experiment will be very informative and we are grateful to the referee for suggesting it. We will inhibit BLM expression alone or together with LIG3 in TRF2-deficient MEFs and quantify telomere fusion frequency. This will help establish whether the observed increase in telomere joining triggered by BLM depletion in TRF2-lacking cells (Fig. 5A) is due to additional activation of A-NHEJ in these cells.

9. μ symbol is messed up in several places in materials and methods.

Response: This will be corrected.

Referee #3:
We would like to thank this referee for the helpful suggestions and relevant literature additions. We will be happy to discuss the papers suggested in the revised version of our manuscript.

1st Editorial Decision 01 July 2014

Thank you for your response to the referee comments on your manuscript, EMBOJ-2014-88947. I have now considered your proposal for addressing the referees' comments and criticisms, and I am pleased to say that after inclusion of these planned revisions, the study would indeed appear to be suited for publication in The EMBO Journal. I would thus like to invite you to prepare and resubmit a new version of the manuscript, revised in response to the referees as suggested. Please keep in mind that our policy to allow only a single round of major revision makes it important to carefully answer to all points during this round, so please let us know in case you should require an extended revision time to achieve this. Finally, please also remember that within our 'scooping protection' policy, competing work published during our agreed revision period will have no negative impact on the final assessment of your study.

Thank you again for the opportunity to consider this work for publication! I look forward to your revision.

1st Revision - authors' response 29 October 2014

Thank you very much for the opportunity to revise our manuscript entitled “BRCA1 and CtIP promote alternative non-homologous end-joining at uncapped telomeres” by Badie et al. according to the referees’ comments. As major additions to our manuscript we included new data, as requested by Referees 1 and 2, demonstrating that: a) the cancer predisposing BRCA1 C61G mutation known to abolish BRCA1 E3 ubiquitin ligase activity and its interaction with BARD1 is not required for uncapped telomere fusions; b) that BRCA1 inhibition did not have an additional effect when PARP1 was depleted, indicating that both act in the same pathway of uncapped telomere fusion; c) that CtIP inhibition further decreased telomere re-joining in Ku80-deficient MEFs, suggetsing that CtIP acts in a Ku80-independent pathway of uncapped telomere fusion; d) that CtIP is required for resection of DNA double stranded breaks introduced at intra-chromosomal sites and d) that BLM can suppress LIG4-independent fusions of uncapped telomeres. We include below a detailed response on how each issue raised by the referees has been addressed in the revised manuscript, as tentatively outlined in our letter of June 25, 2014.

We are grateful for the insightful and generally supportive comments of the referees.
Point-by-point response:

Referee #1:
In this work, Badie and colleagues show that the tumour suppressor protein BRCA1 and its interacting partner CtIP facilitate joining of uncapped telomeres. TRF2 inactivation in mouse embryonic fibroblasts (MEFs) results in DNA damage signalling at telomeres, removal of the 3' single-stranded telomeric overhang and telomeric end-to-end fusions. The data presented here prove that loss of BRCA1 or CtIP does not affect the activation of the DNA damage response and the degradation of the 3' overhang, however it does reduce the frequency of telomere fusions induced by telomere uncapping. Both classical (C-NHEJ; Ku70/80- and LIG4-dependent) and alternative non-homologous end-joining (A-NHEJ; Ku70/80- and LIG4-independent) contribute to telomere fusions. Interestingly, Badie and colleagues demonstrate that CtIP is synergistic to LIG4 but epistatic to LIG3, indicating that CtIP promotes joining of uncapped telomeres through A-NHEJ. Finally, the authors place EXO1 in the same LIG3-dependent A-NHEJ pathway of telomere end-joining.

Overall, the data presented by Badie and colleagues provide useful information to understand how cells process dysfunctional telomeres and demonstrate a substantial contribution of A-NHEJ in the generation of telomere fusions. Given that there is an increasing interest in understanding A-NHEJ in a variety of contexts, this work will certainly be of interest to the readership of EMBO Journal.

That being said, some of the results presented in this manuscript are in apparent contradiction to previously published data, primarily from the de Lange lab (in particular, the seminal Sfeir et al. 2012 Science). While, I understand that the experimental systems are different, I feel that it is critical that the authors provide additional controls/experiments to firm up the proposed model shown in Figure 6.

Secondly, since it is practically impossible to sequence telomeric fusion events and infer the type of DNA repair events, the manuscript is largely based on genetic and knockdown analyses. With this in mind, I would define A-NHEJ as Ku- and Lig4-independent and end-joining. Since Ku also actively suppresses A-NHEJ, it is easier to study A-NHEJ in contexts of Ku deficiency. The manuscript would have been stronger if some of the analyses had been carried out in Ku-deficient MEFs.

Response: We agree with the referee’s view that there are fundamental differences between our system based on TRF2 shRNA-mediated depletion and that used by Sfeir et al. (2012) in which concomitant deletion of *Trf1* and *Trf2* genes caused removal of the entire shelterin complex from the telomeres. On page 4, last paragraph, we have addressed the discrepancies between the two studies as suggested by the referee.

We emphasize in the manuscript that the A-NHEJ events we monitor are defined as Ku80- and LIG4-independent (e.g. page 8, bottom paragraph).

As suggested by the referee, we have repeated some of the analyses in *Ku80*-deficient MEFs. The new data presented in Figures 3E, F (discussed on page 8, middle paragraph) demonstrate that CtIP inactivation in *Ku80*-deficient MEFs causes a further decrease in telomeric

fusions, similarly to its effect in *Lig4*-deficient MEFs (Figures 3C, D). In addition (and in response to referee's Point 2), in Supplementary Figure S3 we now present evidence that BRCA1 inactivation in *Lig4*$^{-/-}$ MEFs also has an additive effect on fusion frequency, similarly to the effect of CtIP depletion presented in Figures 3C, D.

Specific points:

1. As the authors surely know, the effect of BRCA1 on DNA end-resection is somewhat controversial, and so is its requirement for CtIP activation, ubiquitylation and recruitment to DNA damage sites. The model proposed by the authors would be much stronger if a structure-function analysis was carried out to test the requirement of BRCA1 E3 ligase activity in this process or its interaction with CtIP in mediating fusions at uncapped telomeres.

Response: Following referee's suggestion, we examined the contribution of BRCA1 E3 ligase activity to telomere fusion formation. We used knock-in MEFs carrying the *Brca1*^{C61G} mutation (generated in Dr. Jos Jonkers' laboratory) which disrupts the BRCA1 RING domain, abrogate its E3 ubiquitin ligase activity and its interaction with BARD1 (Drost et al., 2011). We found no effect of the C61G mutation on the frequency of telomere fusions triggered by TRF2 depletion, as shown in the new Figure 3B (discussed on page 8, top paragraph). We have attempted to address whether the interaction between BRCA1 and CtIP is required at telomeres by expressing a CtIP S327A mutant lacking the CDK-dependent phosphorylation site required for BRCA1/CtIP interaction, in TRF2-deficient and Cre-treated *Ctip*$^{F/-}$ immortalized MEFs. However, our attempts to establish expression of this protein in MEFs using various expression vectors (e.g. pEGFP-C1 or pEF1A or pBabe) were not successful, due to its toxicity in this type of cells.

2. Figure 3-C shows that CtIP and LIG4 independently contribute to telomeric fusions, which is a strong argument to suggest that CtIP participates in A-NHEJ. Is this also true for BRCA1?

Response: In order to address the referee's question, we have established effective shRNAs targeting mouse BRCA1 and depleted this protein together with TRF2 in *Lig4*$^{-/-}$ MEFs. These results, now presented in the new Supplementary Figure S3, demonstrate that BRCA1 inhibition further reduced fusion levels in *Lig4*-negative MEFs.

3. As mentioned above, the authors speculate that the overlapping requirement for LIG3 and LIG4 for telomere end-joining could be explained if the two act together in certain ligation events. If this is the case, then using LIG3 and LIG4 as a means to distinguish between C-NHEJ and A-NHEJ is not appropriate. The authors should test the contribution of BRCA1 and CtIP in conditions where either Ku70/80 or PARP1 are genetically deleted/depleted as a means to support their conclusions.

Response: As discussed above, CtIP depletion in *Ku80*$^{-/-}$ MEFs (new Figures 3E, F) caused a further reduction in fusion frequency, indicating that CtIP is required for Ku80-independent (as well as LIG4-independent in Figures 3C, D) telomere joining. We did not have access to *Parp1*$^{-/-}$ MEFs to perform a similar experiment. Instead, we inhibited PARP1 expression using lentivirus-delivered shRNA (as reported by Sfeir et al., 2012) together with TRF2 depletion in Cre-treated *Brca1*$^{F/-}$ MEFs. These results, now presented in the new Figures 4D, E, demonstrate that BRCA1 and PARP1 act in the same pathway of uncapped telomere fusion.

4. It was previously reported that the increased resection observed in 53BP1-deficient cells is mediated, at least in part, by CtIP not only at dysfunctional telomeres (Sfeir and de Lange, 2012) but also at intra-chromosomal double-strand breaks (Bothmer et al, 2013). However the results presented in Figure 4 contradict this previously published data. Since these results may generate confusion in the field, one additional control I would suggest to carry out would be to test whether depletion of CtIP is required for resection of intra-chromosomal DSBs in conditions of 53BP1 depletion in the cells used for Figure 4. In other words, the authors should see if they can see the role of CtIP in end-resection at non-telomeric sites in the same cells used to determine the function of CtIP in ssDNA generation at dysfunctional telomeres.

Response: This is a great suggestion for a control that would demonstrate that genome-wide break resection relies on CtIP. In the new Supplementary Figure S4 we have monitored end-resection at non-telomeric sites using ionizing radiation-induced RAD51 focus formation as a readout (as described by Polato et al., 2014). In MEFs lacking 53BP1 and/or CtIP the frequency of RAD51 foci decreased significantly upon CtIP inactivation, indicating that CtIP is required for resection of intra-chromosomal DSBs (discussed on page 10, top paragraph).

> *5. BRCA1 and CtIP participate in DSB repair pathway choice by suppressing RIF1 in the S and G2 phases of the cycle (work by the Boulton, Durocher and Chen labs). One possibility that has not been considered by the authors is that RIF1 blocks A-NHEJ and that BRCA1 and CtIP can relieve this inhibition. This model might be difficult to address using RNAi-mediated depletions but is certainly worth discussing.*

Response: We are grateful for this suggestion. Indeed we do not have access to *Rif1*$^{-/-}$ MEFs, but have included a discussion of the potential roles of RIF1 in telomere fusions on pages 13 and 16, bottom paragraphs.

Referee #2:

In this manuscript Badie et al., used TRF2 shRNA mediated depletion in BRCA1 null and CtIP shRNA depleted cells to investigate the role of BRCA1 and CtIP in A-NHEJ. Deletion of BRCA1 and CtIP in TRF2 depleted cells leads to significant reduction in chromosome fusions as compared to TRF2 shRNA alone. The investigators further demonstrate that BRCA1, CtIP and EXOIII act in the same pathway as LIG3 to promote A-NHEJ at TRF2 depleted telomeres. Interestingly, both BRCA1 and CtIP do not function in the ATM-dependent telomere damage signaling, or in telomere overhang removal, which are critical for telomere fusions by C-NHEJ. BRCA1 and CtIP abrogation also leads to an increased frequency of chromatid fusions in TRF2-depleted cells, suggesting that these factors are required in postreplicative processing of both lagging and leading telomere strands at uncapped telomeres. Unfortunately, the present manuscript suffers from a lack of novelty. Both the de Lange and Chang labs already demonstrate that TRF2 loss promotes A-NHEJ repair at telomeres, and that CTIP is involved in this process. In addition, the role of BRCA1 (Zhong et al., J Biol Chem; 2002)has already been demonstrated to be required in A-NHEJ.

Response: We consider that our work is novel for several reasons: 1) Both the de Lange and Chang laboratories have shown that CtIP functions in uncapped telomere resection and A-NHEJ respectively, specifically in cells lacking 53BP1. Here we address the role of CtIP and BRCA1 in cells in which TRF2 is depleted and 53BP1 is functional, a context which resembles physiological cell senescence/ageing. 2) In the work of Titia De Lange and colleagues (Sfeir et al., 2012) the entire shelterin complex dissociates from telomeres through concomitant genetic deletion of *Trf1* and *Trf2*. These are fundamental differences from our experimental system, in which TRF2 is removed from telomeres whilst 53BP1 remains intact. 3) No role for BRCA1 in processing dysfunctional telomeres has been reported so far. 4) The work by Zhong et al. (J Biol Chem, 2002) indicates that *Brca1*-deleted MEFs are defective in microhomology-mediated end joining (MMEJ), assessed at an I-SceI –induced DSB. No epistatic analyses with LIG3 or PARP1 were reported, to definitely place BRCA1 in the A-NHEJ repair pathway. As such, this work can hardly be considered to have assigned an indisputable role for BRCA1 in A-NHEJ.

1. *The authors should use arrow to indicate total and phosho-Chk2 bands in Figure 1A.*

Response: We have added arrows to label total and phospho-CHK2 bands in the WB in Figure 1A.

2. *Figure 2B suggests that chromosome fusions upon removal of TRF2 in BRAC1 and CtIP depleted cells are reduced to 8.2% and 6.6% as compared to 17.6% in wild type cells. The authors should clarify in the text that residual fusions in BRCA1 and CtIP deleted cells represent C-NHEJ fusions.*

Response: We included this clarification on page 6, bottom paragraph.

3. *The authors should reconstitute BRCA1 and CtIP over expressing constructs in TRF2 depleted cells to show the specificity of knockdown.*

Response: In most of our experiments, BRCA1 expression was not reduced by shRNA knockdown, but through conditional *Brca1* deletion using Cre recombinase. Cells treated with vector alone were used as a control. The CtIP shRNA was previously used by Sfeir et al. (2012) and Bunting et al. (2010).

4. *It would be interesting to know which domain of BRCA1 and CtIP are involved in TRF2 depleted A-NHEJ chromosome fusions.*

Response: For a discussion, please see Point 1, Referee no. 1 (above).

5. *BRCA1S988 promotes error-free NHEJ and suppress A-NHEJ (Dueva and Iliakis, Translational Cancer Research; 2013). To gain mechanistic insights into the roles of BRCA1 in A-NHEJ at TRF2 depleted cells, the authors should investigate the function of BRCA1S988 in TRF2 depleted cells.*

Response: In human cells, CHK2-dependent phosphorylation of BRCA1 at Ser988 was reported to be required for effective homologous recombination repair (Zhang et al., Mol. Cell Biol. *24*, 2004). However, there is no indication in the literature that the same residue is phosphorylated in mouse or that this phosphorylation variant is functional in mouse cells.

6. *In addition to LIG3, PARP1 also know to promote A-NHEJ. The authors should deplete PARP1 to further strengthen their data suggesting that BRCA1 and CtIP act in the same pathway as LIG3/PARP1 to promote A-NHEJ at TRF2 depleted telomeres.*

Response: This has been addressed as described in Point 3, Referee no. 1 (above).

7. *Figure 4. suggests that CtIP is not required for the removal of 3' overhang at uncapped telomeres. Does deletion of BRCA1 leads to a similar phenotype?*

Response: We have attempted on several occasions to detect single-stranded DNA in Cre-treated *Brca1*$^{F/-}$ MEFs, but did not succeed probably due to low numbers of cells recovered after the Cre treatment. However, in Supplementary Figure S5 we included new data showing that the reduction in fusion frequency by 53BP1 shRNA treatment is not reversed when *Brca1* is concomitantly deleted using Cre treatment. This suggests that the long overhang generated in the absence of 53BP1 persists in *Brca1*-deleted cells, similarly to CtIP inactivation shown in Figure 5C (discussed on page 10, top paragraph).

8. *Figure 5A suggests that BLM, WRN and DNA2 inactivation together with removal of TRF2 lead to elevated level of telomere fusions, suggesting that these factors act to suppress telomere end-joining. BLM/DNA2 is known to prevent A-NHEJ during DSB repair (Karanja et al., Cell Cycle 2014; Grabarz et al., Cell Report 2013). It would be interesting to know whether these fusions are mediated by C-NHEJ or A-NHEJ.*

Response: We agree with the referee that this experiment is very informative and are grateful for the suggestion. To address whether the elevated fusions are due to C-NHEJ or A-NHEJ, we inhibited BLM expression together TRF2 in *Lig4*-deleted MEFs (new Figure 6B and new Supplementary Figure S8). This caused an increase in telomere fusion frequency compared to TRF2 inhibition alone in *Lig4*-deleted MEFs, suggesting that BLM depletion leads to de-repression of A-NHEJ in TRF2-lacking cells (discussed on page 11, top paragraph). However we could not directly examine the role of LIG3, because concomitant inactivation of LIG3, BLM and TRF2 led to cell cycle arrest, which prevented mitotic entry and fusion analysis.

9. *µ symbol is messed up in several places in materials and methods.*

Response: This has been corrected throughout the manuscript.

Referee #3:

We would like to thank this referee for the helpful suggestions and relevant literature additions. The specific points raised have been addressed as follows:

-Page 3, line 5 from bottom. "Citation of KU-independent NHEJ": the following citations might be quoted for the KU-independent end-joining pathway in mammalian cells: {Feldmann et al., 2000, Nucleic Acids Res, 28, 2585-2596; Guirouilh-Barbat et al., 2004, Mol Cell, 14, 611-623}.

Response: Both references have been included on page 3, lines 6-7 from bottom.

- Page 3, line 2 from bottom, the involvement of PARP1: this paper might be quoted: {Audebert et al., 2004, J Biol Chem, 279, 55117-55126}. This paper is quoted later in the manuscript.

Response: This reference has been included on page 3, line 3 from bottom.

- Page 4, line 2: "The role of CtIP". This reference might be added: {Bennardo et al., 2008, PLoS Genet, 4, (it is cited later in the manuscript).

Response: This reference has been included on page 4, line 2 from top.

- Page 4, {section sign}2, line 7. About MRE11, this paper could be added {Buis et al., 2008, Cell, 135, 85-96}.

Response: This reference has been included on page 4, line 12 from top.

- Page 4, end {section sign}2 (and discussion...). BRCA1 has been shown to interact with KU and to protect against A-NHEJ {Jiang et al., 2013, J Biol Chem}; this corresponds to an opposite role to CtIP. Could the authors comment this "duality".

Response: This is an interesting point; reference and brief discussion have been included on page 13, line 1 from top.

- Page 6/ Could the authors comment (and speculate in discussion) about the differential effects between MRE11 and CtIP.

Response: Brief discussion has been included on page 13, top paragraph.

- Page 8: Since the ablation of KU and XRCC4 lead to different effects on the efficiency of end-joining {Guirouilh-Barbat et al., 2007, Proc Natl Acad Sci U S A, 104, 20902-7}, it would be interesting to know whether it is similar at telomeres (or at least comment).

Response: This reference has been included on page 14, line 15 from top, along with a discussion of the differential effects of XRCC4 and Ku. The impact of Ku80 deletion on uncapped telomere joining is addressed experimentally in new Figures 3E, F.

- Page 8 {section sign}2, CtIP and A-EJ. The following papers (which are quoted later) can also be cited here: {Bennardo et al., 2008, PLoS Genet, 4, e1000110; Grabarz et al., 2013, Cell Rep, 5, 21-8}.

Response: Both references have been included on page 9, lines 6-7 from top.

- Page 9 {section sign}2: 53BP1 counteract resection at DSB: {Grabarz et al., 2013, Cell Rep, 5, 21-8} could be also quoted.

Response: This reference has been included on page 9, line 2 from bottom.

- Page 10, line 3, {Bennardo et al., 2008, PLoS Genet, 4, e1000110} could also be quoted.

Response: This reference has been included on page 11, line 10 from top.

- Page 11{section sign}3, line 6. Ma et. al. is a yeast paper. This has been also described in

mammalian cells {Guirouilh-Barbat et al., 2007, Proc Natl Acad Sci U S A, 104, 20902-7; Guirouilh-Barbat et al., 2004, Mol Cell, 14, 611-623}

Response: We added this reference on page 12, line 8 from bottom.

- Page 13{section sign}1. Different effects of KU and Lig4 on DSB repair efficiency. This has been described (and should be quoted) for regular DSB {Guirouilh-Barbat et al., 2007, Proc Natl Acad Sci U S A, 104, 20902-7; Schulte-Uentrop et al., 2008, Nucleic Acids Res, 36, 2561-9}, consistently with the viability in mice {Karanjawala et al., 2002, DNA Repair (Amst), 1, 1017-26}. These hypotheses should be discussed with regard to the data presented here.

Response: This discussion (together with suggested references) is now included on page 14, paragraph starting on line 4 from top.

Acceptance letter 25 November 2014

Thank you for submitting your revised manuscript for our consideration. It has now been seen once more by the original referees (see comments below), and I am happy to inform you that they are fully satisfied with the revisions and that there are no further objections towards publication in The EMBO Journal.

Referee #1

I am satisfied with the revised version of this manuscript.

Referee #2

The authors have satisfied my concerns.

Referee #3

the authors have adequately addressed my comments

© European Molecular Biology Organization
